# Alkaloid Enantiomers from the Roots of *Isatis indigotica*

**DOI:** 10.3390/molecules24173140

**Published:** 2019-08-29

**Authors:** Dongdong Zhang, Yanhong Shi, Rui Xu, Kang Du, Fujiang Guo, Kaixian Chen, Yiming Li, Rui Wang

**Affiliations:** 1 School of Pharmacy, Shanghai University of Traditional Chinese Medicine, Shanghai 201203, China; 2 Institute of TCM International Standardization of Shanghai University of Traditional Chinese Medicine, Shanghai 201203, China; 3 Shanghai Institute of Materia Medica, Chinese Academy of Science, Shanghai 201203, China

**Keywords:** *Isatis indigotica*, alkaloid enantiomers, structure deduction, anti-inflammatory activity

## Abstract

Five pairs of alkaloid enantiomers (**1a/1b**–**5a/5b**) were obtained from *Isatis indigotica* (*I. indigotica*) roots. Among them, **1a/1b, 2a/2b** and **3a/3b** were determined as three pairs of new alkaloid enantiomers. Their structures were elucidated by physicochemical properties and spectroscopic methods. The absolute configurations were deduced by comparison of their experimental circular dichroism (CD) and calculated electronic circular dichroism (ECD) spectra, as well as by single-crystal X-ray crystallography using anomalous scattering of Cu K*α* radiation. Alkaloids **1a** and **1b** possess an unpresented carbon skeleton and their putative biosynthetic pathways are discussed. Moreover, all of the alkaloids were tested for their nitric oxide (NO) inhibitory effects in RAW 264.7 cells, and **4a** and **4b** showed inhibitory effects with IC_50_ values of 76.97 μM and 65.88 μM, respectively.

## 1. Introduction

**Figure 1:**
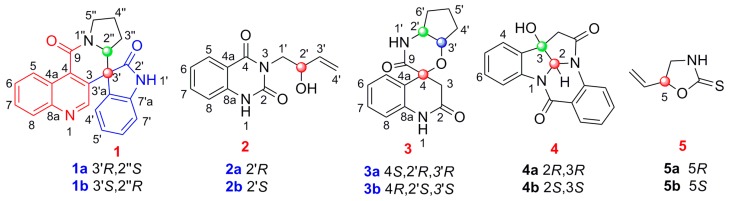
Structures of compounds **1a/1b**–**5a/5b**.

“Ban lan gen”, widely distributed and cultivated in the North of China, is the roots of *Isatis indigotica* Fort. (Cruciferae) [1,2,3]. As one of the most famous traditional Chinese medicines (TCMs), ban lan gen is usually used for the treatment of various kinds of diseases, such as influenza, fever, epidemic hepatitis and infections [4,5,6]. Previous phytochemical investigations of *I. indigotica* have led to the isolation of various kinds of natural constituents, including alkaloids, lignans, flavonoids and nucleotides [1,2,3,4,5,6,7,8,9], among which alkaloids have been considered as the most active constituents that possess anti-inflammatory, antiviral, antibacterial, antitumor and antioxidant activities [1,4,5,6,7,8,9]. For our continuous project to explore more bioactive components from *I. indigotica* [8,9], five pairs of alkaloid enantiomers were isolated from the 80% alcohol exact of *I. indigotica* roots. Among them, **1a/1b**, **2a/2b** and **3a/3b** were determined as three pairs of new enantiomers, whose structures and absolute configurations were determined by extensive spectroscopic data analysis, including 1D, 2D-NMR and HRESIMS data, optical rotation data, comparison of their experimental circular dichroism (CD) and calculated electronic circular dichroism (ECD) spectra and single-crystal X-ray crystallography using anomalous scattering of Cu K*α* radiation. The known forms (**4a/4b**–**5a/5b**, Figure 1) were identified by comparison of their spectroscopic and optical rotation data with those reported in the literature as (−)-(2*R*,3*R*)-3-hydroxy-2*H*-pyrrolo[2-b]indolo[5,5a,6-b,a]quinazoline- 9(8*H*),7′-dione (**4a**) [1], (+)-(2*S*,3*S*)-3-hydroxy-2*H*-pyrrolo[2-b]indolo[5,5a,6-b,a]quinazoline-9(8*H*),7′-dione (**4b**) [1], epigoitrin (**5a**) [10] and goitrin (**5b**) [10]. Alkaloids are one of the main types of active constituents in *I. indigotica*, and they have been reported to possess potential anti-inflammatory effects [1,2,3,4,5,6,7,8,9,10]. This pharmacological action, together with the traditional use for the treatment of epidemic hepatitis, prompted us to test the inhibitory effects on nitric oxide (NO) production of all the isolated alkaloids (**1a/1b**–**5a/5b**). Herein, the isolation and structure elucidation, putative biosynthetic pathways and the NO inhibitory activities of these enantiomers are presented.

## 2. Results and Discussion

**Figure 2:**
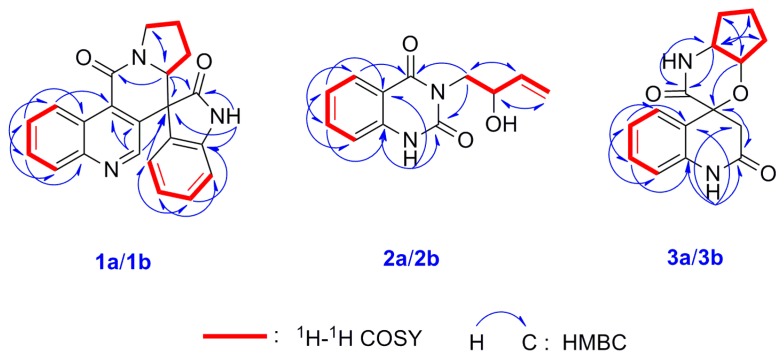
Key ^1^H-^1^H COSY and HMBC correlations of compounds **1a/1b**–**3a/3b**.

**Figure 3:**
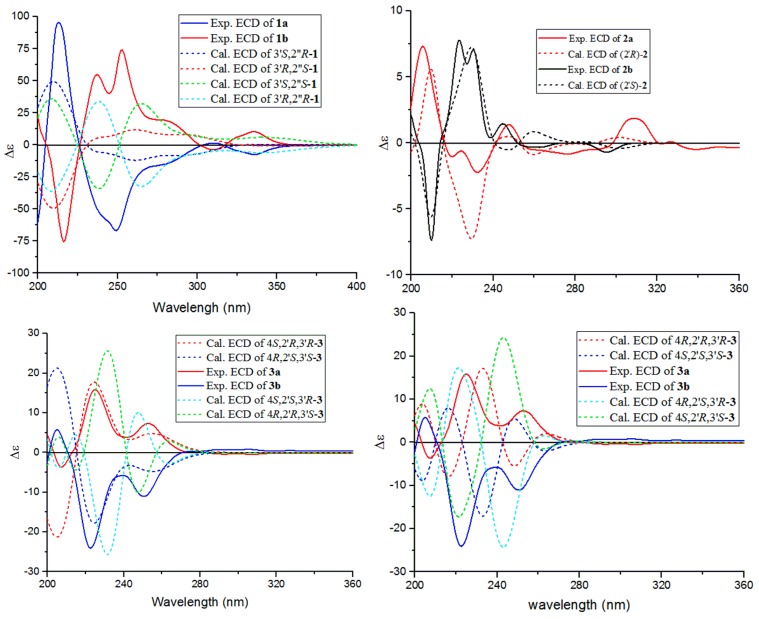
Experimental and calculated electronic circular dichroism (ECD) spectra of compounds **1a/1b**–**3a/3b**.

**Figure 4:**
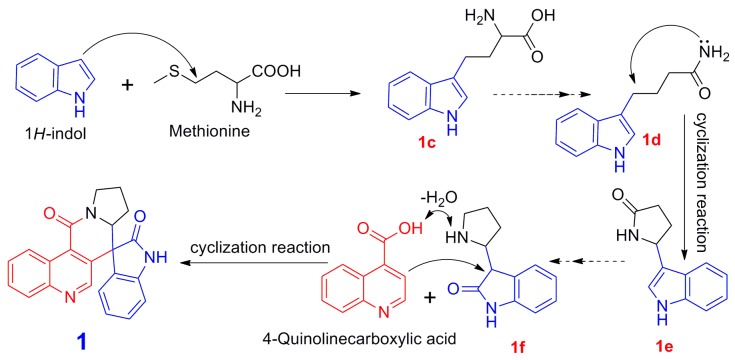
Putative biosynthetic pathway of compound **1**.

Isatisindigoticanine B (**1**) was obtained as a primrose yellow amorphous power. Its molecular formula was determined as C_22_H_17_N_3_O_2_, by the ^13^C NMR data and the HRESIMS quasimolecular ion peak at *m*/*z* 356.1398 [M + H]^+^ (cacld. 356.1394 [M + H]^+^). The ^1^H NMR spectrum (Table 1) of **1** showed signals of two ortho-disubstituted benzene ring at [*δ*_H_ 9.29 (1H, d, *J* = 8.6 Hz, H-5), 7.77 (1H, dd, *J* = 8.6, 7.7 Hz, H-6), 7.84 (1H, dd, *J* = 8.3, 7.7 Hz, H-7) and 8.07 (1H, d, *J* = 8.3 Hz, H-8)] and [6.76 (1H, d, *J* = 7.5 Hz, H-4′), 6.95 (1H, dd, *J* = 7.7, 7.5 Hz, H-5′), 7.30 (1H, dd, *J* = 7.8, 7.7 Hz, H-6′) and 7.05 (1H, d, *J* = 7.8 Hz, H-7′)]; a trisubstituted double bond at *δ*_H_ 8.56 (1H, s, H-2) and an exchangeable proton at *δ*_H_ 11.25 (1H, brs, NH-1′). The ^13^C NMR spectrum (Table 1) displayed 22 carbon signals, and based on the DEPT 135° experiments, 9 × C signals at *δ*_C_ (176.8, 161.6, 148.5, 141.9, 133.4, 132.1, 128.2, 124.6, 55.4), 10 × CH signals at *δ*_C_ (148.3, 130.4, 130.1, 129.8, 128.9, 126.7, 124.4, 123.1, 111.2, 61.8) and 3 × CH_2_ signals at *δ*_C_ (46.6, 27.1, 22.8) were observed. These spectroscopic features, along with the molecular formula and the degrees of unsaturation (16 index of hydrogen deficiency, IHD), suggested that isatisindigoticanine B was an unusual alkaloid [1,2,3,6,7,8,9]. This inference was confirmed by detailed analysis of the 2D NMR data. The proton and protonated carbon resonances in the NMR spectra of **1** were unambiguously assigned by the HSQC experiments [11,12,13,14]. The ^1^H-^1^H COSY correlations of H-4′/H-5′/H-6′/H-7′, along with HMBC correlations (Figure 2) of NH-1′/C-2′, C-3′ and C-7′a, indicate a 1*H*-indol-2-one unit in **1** [15]; ^1^H-^1^H COSY correlations of H-2″/H-3″/H-4″/H-5″, along with the HMBC correlations of H-2″/C-3″ and C-5″, indicated a pyrrolidine unit in **1** [16]; ^1^H-^1^H COSY correlations of H-5/H6/H7/H8, along with the HMBC correlations of H-2/C-3 and C-4, H-5/C-4 and C-8a and the remaining molecular formula C_10_H_5_NO, indicated a 4-quinolinecarboxylic acid unit in **1** [17]. HMBC correlations of H-2/C-3′, C-3 and C-4 and correlations of H-2″/C-9, C-2′, C-3′ and C-5″, determined the 1*H*-indol-2-one unit connected with the pyrrolidine unit and the 4-quinolinecarboxylic acid via a six-membered ring of C-3-C-4-C-9-N-1″-C-2″-C-3′. The planar structure of **1** was thus deduced as depicted in Figure 1. Subsequent HPLC separation of **1** on a chiral column yielded **1a** and **1b** (Figure S34, Supplementary Information) with opposite optical rotations (+12.6° for **1a** and −12.5° for **1b**) and mirrored CD spectra curves (Figure 3). To further determine the absolute configurations of **1**, the ECD curves were simulated for the four epimers of **1**, [(3′*R*,2″*S*)-**1**, (3′*S*,2″*R*)-**1**, (3′*R*,2″*R*)-**1** and (3′*S*,2″*S*)-**1**] (Figure 3). The experimental CD spectra of **1a** and **1b** were well matched with the calculated ECD curves of (3′*R*,2″*S*)-**1** and (3′*S*,2″*R*)-**1**, respectively. Accordingly, the structure of **1a** and **1b** were elucidated as depicted (Figure 1) and named as (+)-(3′*R*,2″*S*)-isatisindigoticanine B (**1a**) and (−)-(3′*S*,2″*R*)-isatisindigoticanine B (**1b**). This carbon skeleton is the first report from a natural source and the putative biosynthetic pathways were proposed (Figure 4).

**Figure 5:**
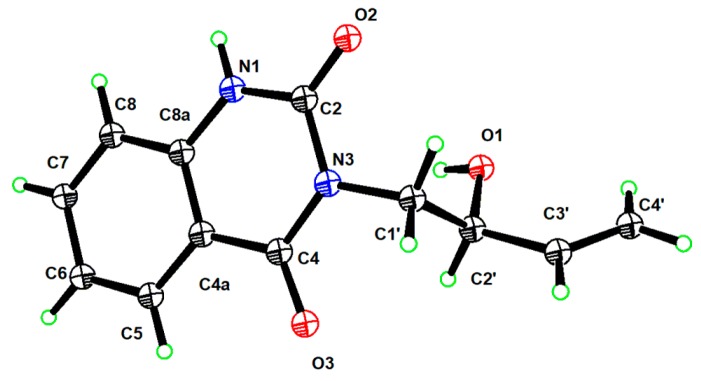
ORTEP darning of (+)-(2′*S*)-isatisindigoticanine C (**2b**).

Isatisindigoticanine C (**2**) was obtained as a white amorphous power. Its molecular formula was assigned as C_12_H_12_N_2_O_3_ by the 1D NMR data and the HRESIMS quasimolecular ion peak at *m*/*z* 231.0771 [M − H]^−^ (cacld. 231.0775 [M − H]^−^). The ^1^H NMR spectrum (Table 1) of **2** showed signals of an ortho-disubstituted benzene ring at *δ*_H_ 7.93 (1H, d, *J* = 8.0 Hz, H-5), 7.22 (1H, dd, *J* = 8.0, 7.7 Hz, H-6), 7.76 (1H, dd, *J* = 8.2, 7.7 Hz, H-7) and 7.16 (1H, d, *J* = 8.2 Hz, H-8) [8,9,15]; a monosubstituted double bond at *δ*_H_ 5.83 (1H, ddd, *J* = 16.9, 10.7, 6.5 Hz, H-3′), 5.01 (1H, dd, *J* = 10.7, 1.2 Hz, H-4′a) and 5.11 (1H, dd, *J* = 16.9, 1.2 Hz, H-4′b) and an exchangeable proton at *δ*_H_ 11.39 (brs, NH-1) [15]. Analysis of the ^13^C NMR, DEPT 135° and HSQC data (Table 1) of **2**, a 2′-hydroxybut-3′-en-1′-yl (45.6, CH_2_; 69.2, CH; 140.0, CH; 115.5, CH_2_) [4,10] and a quinazoline-2,4(1*H*,3*H*)-dione moiety (150.8, C; 162.6, C; 114.3, C; 127.9, CH; 122.9, CH; 135.4, CH; 115.5, CH; 139.9, C) were observed [1,2,3]. HMBC correlations of H-1′/C-2 and C-4 indicated the 2′-hydroxybut-3′-en-1′-yl unit connected with the quinazoline-2,4(1*H*,3*H*)-dione unit via a N-3-C-1 bond [2,3]. This inference was supported by detailed analysis of the 2D NMR data including HSQC, HMBC (Figure 2) and ^1^H-^1^H COSY (Figure 2) experiments. The planar structure of **2** was thus deduced as depicted in Figure 1. Subsequent HPLC separation of **2** on a chiral column yielded **2a** and **2b** in a ratio of approximately 1:1, with opposite optical rotations (−31.1° for **2a** and +33.1° for **2b**) and cotton effects in their experimental ECD spectra (Figures S29 and S35, Supplementary Information). The comparison of the experimental CD spectra and the calculated ECD spectra of **2a** and **2b** (Figure 3) confirmed the two enantiomers as (−)-(2′*R*)-isatisindigoticanine C (**2a,** Figure 1) and (+)-(2′*S*)-isatisindigoticanine C (**2b**, Figure 1), respectively [2,3]. Finally, a single-crystal X-ray experiment with Cu K*α* radiation analysis confirmed the structure of (+)-(2′*S*)-isatisindigoticanine C (**2b**, Figure 5).

Isatisindigoticanine D (**3**), a white amorphous powder, has the molecular formula of C_15_H_16_N_2_O_3_, which was supported by the positive HRESIMS ion at *m*/*z* 273.1245 [M + H]^+^ (cacld. 273.1239 [M + H]^+^) and 1D NMR data. The ^1^H NMR spectrum (Table 1) of **3** showed signals of an ortho-disubstituted benzene ring at *δ*_H_ 7.27 (1H, d, *J* = 7.3 Hz, H-5), 6.87 (1H, dd, *J* = 7.5, 7.3 Hz, H-6), 7.17 (1H, dd, *J* = 7.7, 7.5 Hz, H-7) and 6.76 (1H, d, *J* = 7.7 Hz, H-8) and two exchangeable protons at *δ*_H_ 12.39 (brs, NH-1) and 10.37 (brs, NH-1′) [1,15]. The ^13^C NMR spectrum (Table 1) displayed 15 carbon signals based on the DEPT 135° experiments, and 5 × C signals at *δ*_C_ (179.9, 174.2, 141.3, 131.7, 71.8), 6 × CH signals at *δ*_C_ (128.5, 123.7, 121.9, 109.4, 68.5, 46.9) and 4 × CH_2_ signals at *δ*_C_ (47.9, 43.3, 31.8, 28.4) were observed. The 2D NMR spectra of **3** showed the ^1^H-^1^H COSY correlations of H-5/H-6/H-7/H-8 and HMBC correlations from NH-1/C-2, C-3, C-4a and C-8a, and those from H-3/C-2 and C-4 indicated a 4-hydroxy-2-oxo-1,2,3,4-tetrahydroquinoline-4-carboxylic acid unit in **3** [18], while ^1^H-^1^H COSY correlations of H-2′/H-3′/H-4′/H-5′/H-6′ indicated a cyclopentanamine unit in **3** [19]. HMBC correlations from NH-1′/C-9 and C-6 and from H-2′/C-4 determined the 4-hydroxy-2-oxo-1,2,3,4-tetrahydroquinoline-4-carboxylic acid unit connected with the cyclopentanamine unit via a six-membered ring of N-1′-C-2″-C-3-*O*-C-4-C-9. The planar structure of **3** was thus determined as depicted in Figure 1. Subsequent HPLC separation of **3** on a chiral column yielded **3a** and **3b** (Figure S36, Supplementary Information) with opposite optical rotations (−65.7° for **3a** and +63.2° for **3b**) and mirrored CD spectra curves (Figure 3). Subsequently, the absolute configurations of **3a** and **3b** were determined by comparison of their experimental and calculated ECD spectra at the b3lyp/6-31g(d) level. As shown in Figure 3, the theoretically calculated ECD of (4*S*,2′*R*,3′*R*)-**3** and (4*R*,2′*S*,3′*S*)-**3** matched well with the experimental CD of **3a** and **3b**, respectively. Thus, the structures of the two enantiomers were given and named as (−)-(4*S*,2′*R*,3′*R*)-isatisindigoticanine D (**3a**, Figure 1) and (+)-(4*R*,2′*S*,3′*S*)-isatisindigoticanine D (**3b**, Figure 1), respectively [2,3].

For our continuous project to explore more anti-inflammatory components from *I. indigotica* [8,9], compounds **1a/1b**–**5a/5b** were tested for their inhibitory effects on the NO production in LPS activated RAW 264.7 cells, a primary indicator in assessing inflammatory activities [20]. The results suggested that only **4a** and **4b** exhibited inhibitory activities, with IC_50_ values of 76.97 μM and 65.88 μM.

Isatisindigoticanine B (**1**) is the first example of a 1*H*-indol-2-one unit connected with a 4-quinolinecarboxylic acid unit and a pyrrolidine unit via a six-membered ring of C-3-C-4-C-9-N-1″-C-2″-C-3′. On the basis of its unique structural features, the putative biosynthetic pathways for isatisindigoticanine B (**1**) are proposed in Figure 4. The biosynthetic precursor of **1** is proposed from 1*H*-indol [4]. First, 1*H*-indol was connected with methionine moiety by sequential or simultaneous enzymatic catalysis to give **1c** [4,5], and then **1c** was modified via an enzyme-catalyzed reaction to give **1d** [20]. Then, **1e** was obtained by cyclization reaction of **1d** [8] and then changed via a reduction reaction to give **1f**, and finally **1f** was modified by steps of dehydration and cyclization reactions [5,8] to give **1**. Compound **1** was separated by chiral analysis to give **1a** and **1b** [2,3].

## 3. Experimental Section

The general experimental procedures and extraction and isolation sections are listed in the Supplementary Information section. The plant material (*I. indigotica* roots) was used in the same way we described previously [8,9].

### 3.1. Physical and Spectroscopic Data of Isatisindigoticanines B–D

Isatisindigoticanine B (**1**), a primrose yellow amorphous power; IR (KBr) *ν*_max_: 3407, 2923, 1720, 1666, 1613, 1501, 1460, 1350, 1267, 1023, 954, 759 cm^−1^; *m*/*z* 356.1398 [M + H]^+^ (cacld. 356.1394 [M + H]^+^); ^1^H NMR (DMSO-*d*_6_, 600 MHz) and ^13^C NMR (DMSO-*d*_6_, 150 MHz) (Table 1); [α]${}_{D}^{20}$ +12.6° (*c* 0.15, MeOH) for (+)-(3′*R*,2″*S*)-isatisindigoticanine B (**1a**) and [α]${}_{D}^{20}$ −12.5° (*c* 0.07, MeOH) for (−)-(3′*S*,2″*R*)-isatisindigoticanine B (**1b**).

Isatisindigoticanine C (**2**), a white amorphous power; IR (KBr) *ν*_max_: 3420, 2939, 1636, 1598, 1514, 1461, 1261, 1139, 1025, 859, 813 cm^−1^; *m*/*z* 233.0771 [M − H]^−^ (cacld. 233.0775 [M − H]^−^); ^1^H NMR (DMSO-*d*_6_, 600 MHz) and ^13^C NMR (DMSO-*d*_6_, 150 MHz) (Table 1); [α]${}_{D}^{20}$ −31.1° (*c* 0.21, MeOH) for (−)-(2′*R*)-isatisindigoticanine C (**2a**) and [α]${}_{D}^{20}$ +33.1° (*c* 0.13, MeOH) for (+)-(2′*S*)-isatisindigoticanine C (**2b**).

Isatisindigoticanine D (**3**), a white amorphous power; IR (KBr) *ν*_max_: 3363, 2924, 1703, 1667, 1514, 1443, 1408, 1260, 1026, 957, 726 cm^−1^; *m*/*z* 273.1245 [M + H]^+^ (cacld. 273.1239 [M + H]^+^); ^1^H NMR (DMSO-*d*_6_, 600 MHz) and ^13^C NMR (DMSO-*d*_6_, 150 MHz) (Table 1); [α]${}_{D}^{20}$ −65.7° (*c* 0.18, MeOH) for (−)-(4*S*,2′*R*,3′*R*)-isatisindigoticanine D (**3a**) and [α]${}_{D}^{20}$ +63.2° (*c* 0.10, MeOH) for (+)-(4*R*,2′*S*,3′*S*)-isatisindigoticanine D (**3b**).

### 3.2. ECD Calculation of Compounds ***1a/1b**–**3a/3b***

The conformers of compounds **1a/1b**–**3a/3b** were obtained using the MM2 force field with ChemBio3D software. Gaussian 09 software was utilized for the semiempirical PM3 quantum mechanical geometry optimizations and the time-dependent density functional theory (TDDFT). ECD was calculated at the b3lyp/6-31g(d) level [20,21,22]. The ECD spectra conformers of **1a/1b**–**3a/3b** were obtained using SpecDis 1.62 and were compared with the experimental data; the calculation details are listed in the supporting information (Figures S26–S33).

### 3.3. X-ray Crystallography of (+)-(2′S)-isatisindigoticanine C (***2b***)

A crystal of **2b** was obtained in MeOH. The crystallographic data of **2b** were obtained with Cu K*α* (λ = 1.54178 Å) radiation at 130 K on a Bruker Apex II CCD diffractometer. The structures were solved by a direct method and refined with the full-matrix least-squares technique using SHELX-2014 software. Nonhydrogen atoms were refined with anisotropic displacement parameters, and hydrogen atoms were placed in calculated positions and refined with a riding model. The flack parameter was 0.15(7) (Figure S37, Supplementary Information). The crystallographic data of **2b** were deposited in the Cambridge Crystallographic Data Centre (CCDC) with deposition number 1941685.

### 3.4. Inhibitory Assay of NO Production

Compounds **1a/1b**–**5a/5b** were tested for their NO inhibitory effects in the LPS activated RAW 264.7 cells using the previously reported method [8,9,23]. The IC_50_ values showed that only **4a** and **4b** showed inhibitory effects with IC_50_ values of 76.97 μM and 65.88 μM (aminoguanidine hydrochloride was used as the positive control, IC_50_ 22.67 μM).

## 4. Conclusions

In this study, six new alkaloids: (+)-(3′*R*,2″*S*)-isatisindigoticanine B (**1a**), (−)-(3′*S*,2″*R*)-isatisindigoticanine B (**1b**), (−)-(2′*R*)-isatisindigoticanine C (**2a**), (+)-(2′*S*)-isatisindigoticanine C (**2b**), (−)-(4*S*,2′*R*,3′*R*)-isatisindigoticanine D (**3a**) and (+)-(4*R*,2′*S*,3′*S*)-isatisindigoticanine D (**3b**), together with four known ones: (−)-(2*R*,3*R*)-3-hydroxy-2*H*-pyrrolo[2,3-b]indolo[5,5a,6-b,a]quinazoline-9(8*H*),7′-dione (**4a**), (+)-(2*S*,3*S*)-3-hydroxy-2*H*-pyrrolo[2-b]indolo[5,5a,6-b,a]quinazoline-9(8*H*),7′-dione (**4b**), epigoitrin (**5a**) and goitrin (**5b**), were isolated from the roots of *I. indigotica*. The alkaloids **1a** and **1b** possess an unpresented carbon skeleton of a 1*H*-indol-2-one unit connected with a 4-quinolinecarboxylic acid unit and a pyrrolidine unit via a six-membered ring of C-3-C-4-C-9-N-1″-C-2″-C-3′. Alkaloids **4a** and **4b** showed NO inhibitory effects in the LPS activated RAW 264.7 cells, with IC_50_ values of 76.97 μM and 65.88 μM.

## Figures and Tables

**Table 1:** ^1^H NMR (600 MHz in DMSO-*d*_6_) and ^13^C NMR data (150 MHz in DMSO-*d*_6_) of **1a/1b**–**3a/3b** (ov: overlap signals).

No.	1a/1b		2a/2b		3a/3b	
	*δ*_H_ (*J* in Hz)	*δ*_C_	*δ*_H_ (*J* in Hz)	*δ*_C_	*δ*_H_ (*J* in Hz)	*δ*_C_
1			11.39, brs		12.39, brs	
2	8.56, s	148.3		150.8		179.9
3		133.4			2.43, 2H, ov	47.9
4		132.1		162.6		71.8
4a		124.6		114.3		131.7
5	9.29, d (8.6)	126.7	7.93, d (8.0)	127.9	7.27, d (7.3)	123.7
6	7.77, dd (8.6, 7.7)	128.9	7.22, dd (8.0, 7.7)	122.9	6.87, dd (7.5, 7.3)	121.9
7	7.84, dd (8.3, 7.7)	130.4	7.76, dd (8.2, 7.7)	135.4	7.17, dd (7.7, 7.5)	128.5
8	8.07, d (8.3)	129.8	7.16, d (8.2)	115.5	6.76, d (7.7)	109.4
8a		148.5		139.9		141.3
9		161.6				174.2
1′	11.25, brs		3.84, dd (12.6, 5.0)	45.6	10.37, brs	
			4.01, dd (12.6, 5.0)			
2′		176.8	4.34, dd (12.6, 6.5)	69.2	3.33, m	46.9
3′		55.4	5.83, ddd (16.9, 10.7, 6.5)	140.0	3.82, dd (7.7, 1.4)	68.5
3′a		128.2				
4′	6.76, d (7.5)	124.4	5.01, dd (10.7, 1.2)	115.5	1.71, m	31.8
			5.11, dd (16.9, 1.2)		1.90, m	
5′	6.95, dd (7.7, 7.5)	123.1			1.73, m; 1.99, m	28.4
6′	7.30, dd (7.8, 7.7)	130.1			2.28, dd (12.5, 6.2)	43.3
					2.43, ov	
7′	7.05, d (7.8)	111.2				
7′a		141.9				
2′’	4.44, dd (9.1, 2.6)	61.8				
3′’	1.11, m; 1.97, m	27.1				
4′’	1.94, 2H, m	22.8				
5′’	3.48, ov; 3.81, m	46.6				
2′-OH			5.12, d (5.2)			

## Supplementary Materials

The following are available online. Copies of ^1^H NMR and ^13^C NMR spectra of **1**–**5**, IR, HREIMS, DEPT 135°, HSQC, HMBC, ^1^H-^1^H COSY spectra of compounds **1**–**3**; ECD calculation details of **1a/1b**–**3a/3b**; Chiral separation chromatography of **1–****5**; Crystallographic data of **2b**.
