# Supplementary material for: Alkaloid Enantiomers from the Roots of Isatis indigotica
Source: Molecules. 2019 Aug 29;24(17):3140. doi: 10.3390/molecules24173140 (PMC6749297; doi:10.3390/molecules24173140)
Supplement: Supplementary file 1 [file molecules-24-03140-s001.pdf]

Supplementary Materials

Alkaloid enantiomers from the roots of *Isatis indigotica*

Dongdong Zhang [1], Yanhong Shi [2], Rui Xu [1], Kang Du [1], Fujiang Guo [1], Kaixian Chen [1, 3],

Yiming Li [1, *] and Rui Wang [1, *]

Affiliation

[1] School of Pharmacy, Shanghai University of Traditional Chinese Medicine, Shanghai, 201203, China

[2] Institute of TCM International Standardization of Shanghai University of Traditional Chinese Medicine, Shanghai, 201203, China

[3] Shanghai Institute of Materia Medica, Chinese Academy of Science, Shanghai, 201203, China

Corresponding Author

[*] E-mail: wr@shutcm.edu.cn (Rui Wang), Tel: 86 21 51322181, Fax: 86 21 51322193.

E-mail: ymlius@163.com (Yiming Li), Tel: 86 21 51322191, Fax: 86 21 51322193.

ORCID: Dongdong Zhang: 0000-0003-0956-1261; Yiming Li: 0000-0003-3416-1331; Rui Wang: 0000-0002-6204- 5015

General experimental procedures

Optical rotation was measured using a Rudolph Autopol VI polarimeter (Rudolph, USA); ECD spectra were obtained on a Applied photophysics brighttime chirascan (AppliedPhotophysics, UK); IR spectra were recorded on a Nicolet iS10 instrument (Thermo Fisher Scientific, USA); 1D and 2D NMR spectra were recorded on a Bruker-Avance 600 instrument (Bruker, Germany); The HR-ESI-MS was performed using a Q-TOF-Ultima mass spectrometer (Milford, MA, USA); The crystallographic data were obtained on a Bruker Apex II CCD diffractometer (Bruker, Germany) using Cu-Kα radiation (λ = 1.54178 Å); Semipreparative HPLC was performed on an Agilent infinity II system equipped with a DAD detector (Agilent, USA) and a Capcell Pak C_{18} column (10 mm × 250 mm, 5μm particles, Shiseido, Japan) or a Chiralpak AD-H column (4.6 mm × 250 mm, 5μm particles, Daicel (China) Investment Co., Ltd.) or ; Sephadex LH-20 (GE Healthcare Bio-Sciences AB); Reversed-phase C_{18} silica gel 5μm, YMC Co., Ltd. Japan); MCI gel (CHP-20 P, Mitsubishi Chemical Industries Co., Ltd. Japan); Silica gel (100–200 mesh and 200–300 mesh; Qingdao Haiyang Chemical, China); All solvents used in CC were of analytical grade (Sinopharm Chemical Reagent Co., Ltd. China).

Extraction and isolation

The air-dried and pulverized root of *I. indigotica* (45 kg) was extracted with 80% EtOH under reflux three times. After removing the solvent under reduced pressure, the concentrated residue was successively partitioned with petroleum ether (PE), dichloromethane (CH_2Cl_2) and *n*-BuOH. The CH_2Cl_2 extract (170 g) was subjected to column chromatography (CC) on silica gel, eluting with a gradient solvent system (CH_2Cl_2-MeOH, 100:0 − 100:20) to give eleven fractions (F1 − F11);

F3 (16g) was subjected to CC on silica gel, eluting with (CH_2Cl_2-MeOH, 100:1 − 100:5) to give six subfractions (F3-1 − F3-6). F3-3 (0.9g) was subjected to CC on Sephadex LH-20 gel, eluting with (CH_2Cl_2-MeOH, 1:1) and then purified by HPLC with MeCN-H_2O (32:68) to afford **5** (200 mg; t_R = 5.8 min). **5** was purified by HPLC with a Chiral pak CD-Ph column, MeCN-H_2O (80:20) to afford **5a** (118 mg, t_R = 17.2 min) and **5b** (45 mg, t_R = 18.8 min); F-4 (14 g) was subjected to CC on silica gel, eluting with (CH_2Cl_2-MeOH, 100:1 ~ 100:5) to give five subfractions (F4-1 − F4-5). F4-3 (1.9g) was subjected to CC on Sephadex LH-20 gel, eluting with (CH_2Cl_2-MeOH, 1:1) and then purified by HPLC with MeCN-H_2O (25:75) to afford **4** (7.9 mg, t_R = 25.5 min), **4** was further purified by HPLC with a Chiral pak AD-H column, normal hexane-isopropanol (18:82) to afford **4a** (2.6 mg, t_R = 16.9 min) and **4b** (2.7 mg, t_R = 15.5 min); F4-4 (0.8g) was subjected to CC on Sephadex LH-20 gel, eluting with (CH_2Cl_2-MeOH, 1:1) and then purified by HPLC with MeCN-H_2O (22:78) to afford **2** (8.5 mg; t_R = 22.6 min), and further purified by HPLC with a Chiral pak AD-H column, normal hexane-isopropanol (80:20) to afford **2a** (3.3 mg, t_R = 27.2 min) and **2b** (3.0 mg, t_R = 24.3 min); F8 (4g) was subjected to CC on RP-C_{18} eluting with MeCN-H_2O (10%, 30%, 60%) to give three subfractions (F8-1 − F8-3). F8-2 (0.2g) was purified by HPLC with MeCN-H_2O (32:68) to afford **3** (10.2 mg, t_R = 13.3 min) further purified by HPLC with a Chiral pak AD-H column, normal hexane-isopropanol (15:85) to afford **3a** (3.8 mg, t_R = 25.1 min) and **3b** (4.1 mg, t_R = 23.9 min); F8-2 (0.4g) was purified by HPLC with MeCN-H_2O (35:65) to afford **1** (6.3 mg, t_R = 14.2 min) further purified by HPLC with a Chiral pak AD-H column, normal hexane-isopropanol (20:80) to afford **1a** (3.8 mg, t_R = 13.0 min) and **1b** (4.1 mg, t_R = 16.1 min).

List of Content

No.	Content	page
1	**Figure S1.** The IR spectrum of **1a/1b** (in KBr)	**S6**
2	**Figure S2.** The HR-ESI-MS spectrum of **1a/1b** (in MeOH)	**S7**
3	**Figure S3.** The ^{1}H NMR spectrum of **1a/1b** (in DMSO-d_6)	**S8**
4	**Figure S4.** The ^{13}C NMR spectrum of **1a/1b** (in DMSO-d_6)	**S9**
5	**Figure S5.** The DEPT 135° spectrum of **1a/1b** (in DMSO-d_6)	**S10**
6	**Figure S6.** The HSQC spectrum of **1a/1b** (in DMSO-d_6)	**S11**
7	**Figure S7.** The HMBC spectrum of **1a/1b** (in DMSO-d_6)	**S12**
8	**Figure S8.** The ^{1}H-^{1}H COSY spectrum of **1a/1b** (in DMSO-d_6)	**S13**
9	**Figure S9.** The IR spectrum of **2a/2b** (in KBr)	**S14**
10	**Figure S10.** The HR-ESI-MS spectrum of **2a/2b** (in MeOH)	**S15**
11	**Figure S11.** The ^{1}H NMR spectrum of **2a/2b** (in DMSO-d_6)	**S16**
12	**Figure S12.** The ^{13}C NMR spectrum of **2a/2b** (in DMSO-d_6)	**S17**
13	**Figure S13.** The DEPT 135° spectrum of **2a/2b** (in DMSO-d_6)	**S18**
14	**Figure S14.** The HSQC spectrum of **2a/2b** (in DMSO-d_6)	**S19**
15	**Figure S15.** The HMBC spectrum of **2a/2b** (in DMSO-d_6)	**S20**
16	**Figure S16.** The ^{1}H-^{1}H COSY spectrum of **2a/2b** (in DMSO-d_6)	**S21**
17	**Figure S17.** The IR spectrum of **3a/3b** (in KBr)	**S22**
18	**Figure S18.** The HR-ESI-MS spectrum of **3a/3b** (in MeOH)	**S23**
19	**Figure S19.** The ^{1}H NMR spectrum of **3a/3b** (in DMSO-d_6)	**S24**
20	**Figure S20.** The ^{13}C NMR spectrum of **3a/3b** (in DMSO-d_6)	**S25**
21	**Figure S21.** The DEPT 135° spectrum of **3a/3b** (in DMSO-d_6)	**S26**
22	**Figure S22.** The HSQC spectrum of **3a/3b** (in DMSO-d_6)	**S27**

23	**Figure S23.** The HMBC spectrum of **3a/3b** (in DMSO-d_6)	**S28**
24	**Figure S24.** The ^{1}H-^{1}H COSY spectrum of **3a/3b** (in DMSO-d_6)	**S29**
25	**Figure S25.** The ROESY spectrum of **3a/3b** (in DMSO-d_6)	**S30**
26	**Figure S28.** b3lyp/6-31g(d) optimized lowest energy conformers for (3'*S*,2"*R*)-**1** and (3'*S*,2"*S*)-**1** and their equilibrium populations	**S31**
27	**Figure S27**. Experimental and calculated ECD spectrum of **1**	**S32**
28	**Figure S28.** b3lyp/6-31g(d) optimized lowest energy conformers for (2'*R*)-**2** and their equilibrium populations	**S33**
29	**Figure S29**. Experimental and calculated ECD spectrum of **1**	**S34**
30	**Figure S30.** b3lyp/6-31g(d) optimized lowest energy conformers for (4*S*,2'*R*,3'*R*)-**3** and (4*S*,2'*S*,3'*R*)-**3** and their equilibrium populations	**S35**
31	**Figure S31.** b3lyp/6-31g(d) optimized lowest energy conformers for (4*R*,2'*R*,3'*R*)-**3** and (4*R*,2'*S*,3'*R*)-**3** and their equilibrium populations	**S36**
32	**Figure S32**. Experimental and calculated ECD spectrum of **3**	**S37**
33	**Figure S33**. Experimental and calculated ECD spectrum of **3**	**S38**
34	**Figure S34.** Chiral separation chromatography of **1**	**S39**
35	**Figure S35.** Chiral separation chromatography of **2**	**S40**
36	**Figure S36.** Chiral separation chromatography of **3**	**S41**
37	**Figure S37.** Crystallographic data of **2b**	**S42**
38	**Figure S38.** The ^{1}H NMR spectrum of **4a/4b** (in DMSO-d_6)	**S43**
39	**Figure S39.** The ^{13}C NMR spectrum of **4a/4b** (in DMSO-d_6)	**S44**
40	**Figure S40.** Chiral separation chromatography of **4**	**S45**
41	**Figure S41.** The ^{1}H NMR spectrum of **1a/1b** (in D_2O)	**S46**
42	**Figure S42.** The ^{13}C NMR spectrum of **1a/1b** (in D_2O)	**S47**
43	**Figure S43.** Chiral separation chromatography of **5**	**S48**

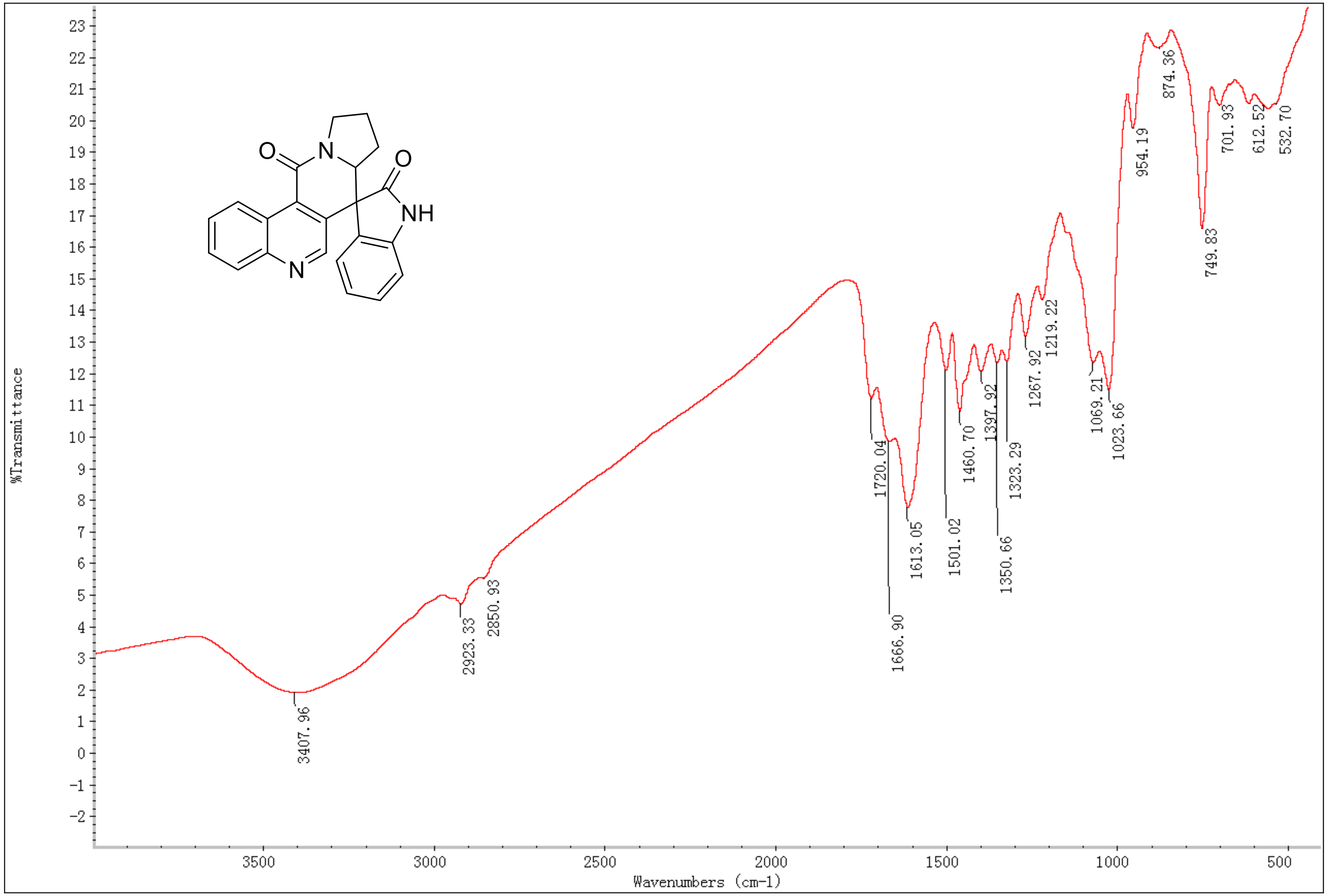

Figure S1. The IR spectrum of **1a/1b** (in KBr)

Qualitative Analysis Report

**Data Filename**	ESIH_20181024_ZWL_ZYT_04.d	**Sample Name**	ZYT-002-42
**Sample Type**	Sample	**Position**	P1-B2
**Instrument Name**	Agilent G6520 Q-TOF	**Acq Method**	20160322_MS_ESIH_POS_1min.m
**Acquired Time**	10/24/2018 19:33:50	**IRM Calibration Status**	Success
**DA Method**	small molecular data analysis method.m	**Comment**	ESIH by ZZY

User Spectra

Fragmentor Voltage	Collision Energy	Ionization Mode
175	0	ESI

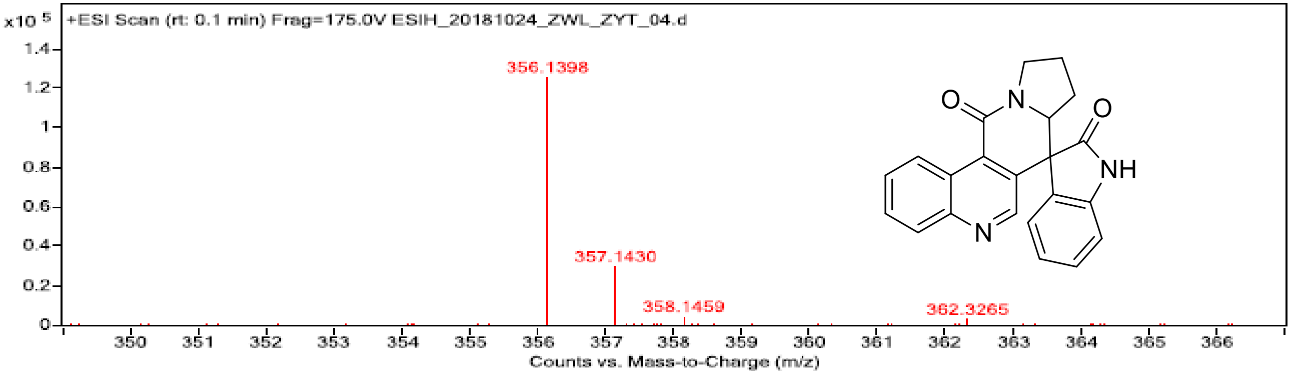

Formula Calculator Results

m/z	Calc m/z	Diff (mDa)	Diff (ppm)	Ion Formula	Ion
356.1398	356.1394	-0.4	-1.12	C22 H18 N3 O2	(M+H)+

--- End Of Report ---

Figure S2. The HR-ESI-MS spectrum of **1a/1b** (in MeOH)

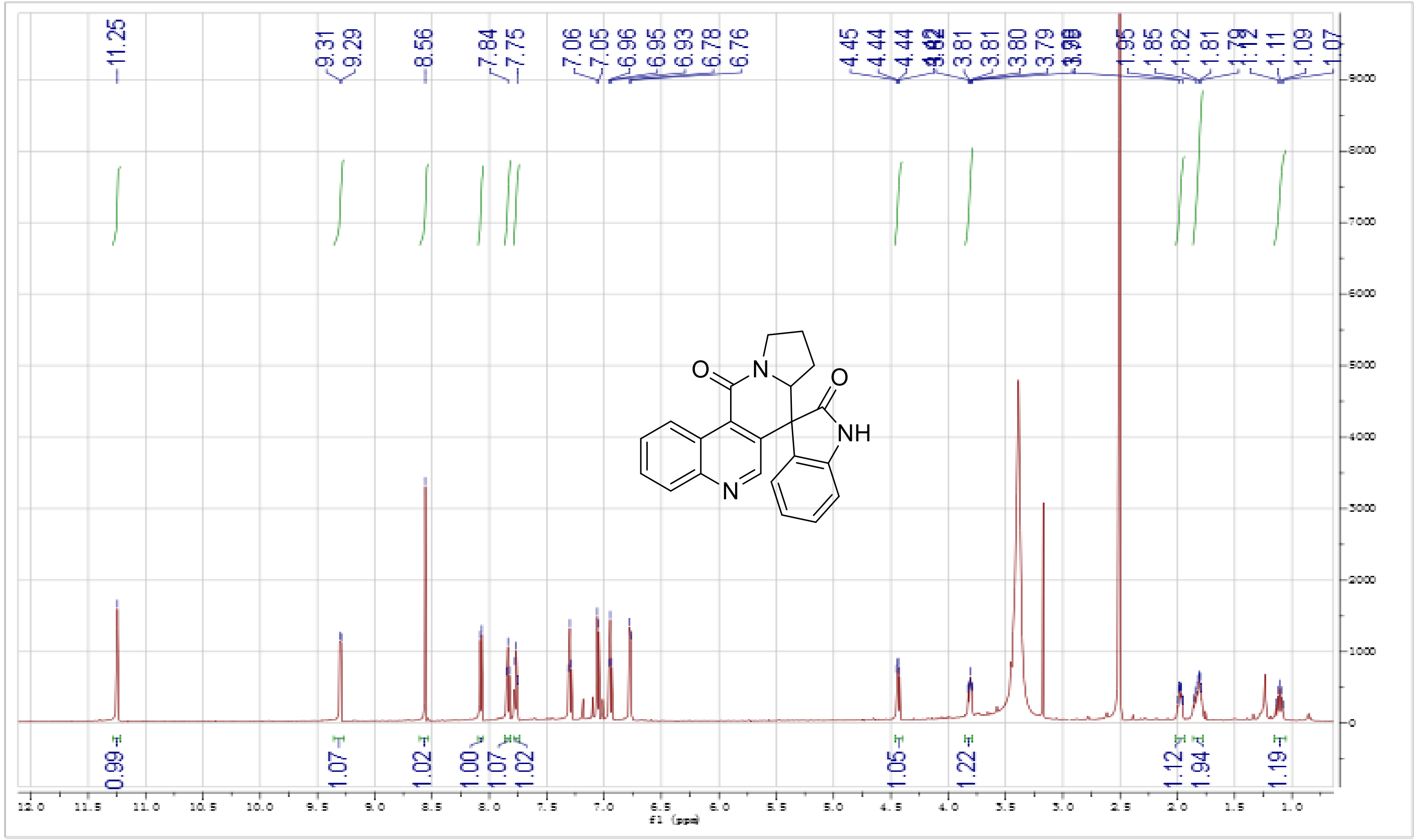

Figure S3. The ^{1}H NMR spectrum of **1a/1b** (in DMSO-d_6)

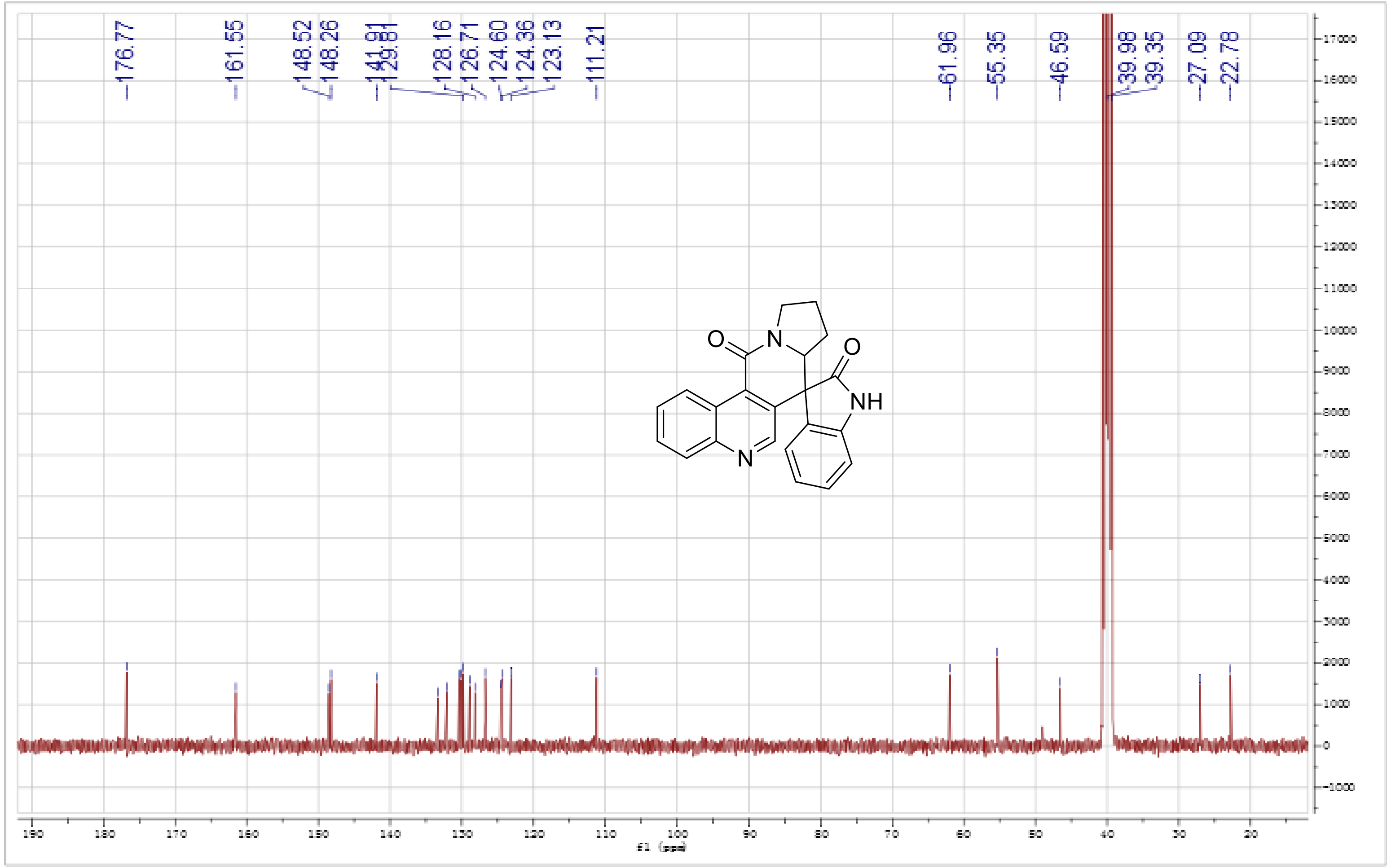

Figure S4. The ^{13}C NMR spectrum of **1a/1b** (in DMSO-d_6)

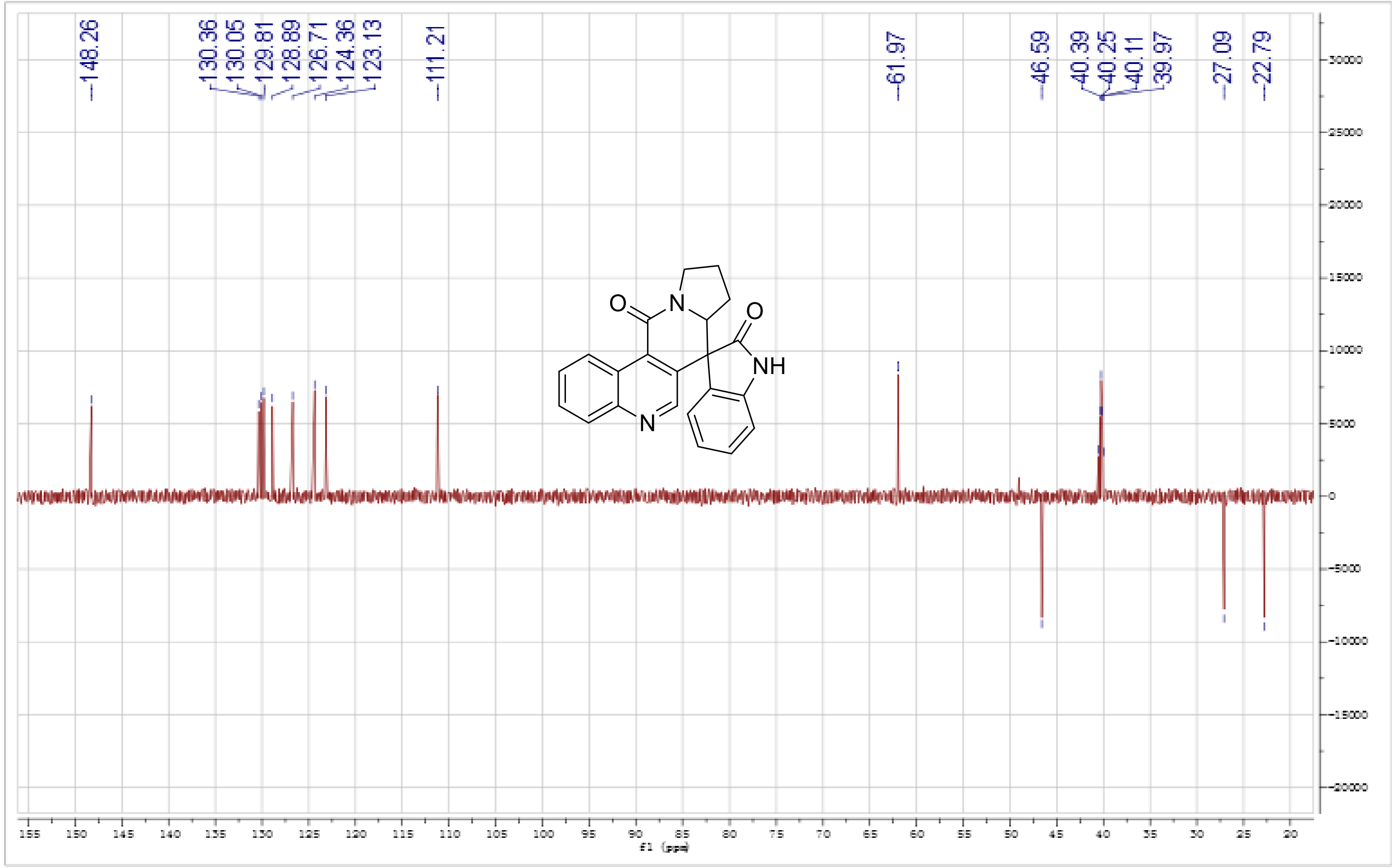

Figure S5. The DEPT 135° spectrum of **1a/1b** (in DMSO-d_6)

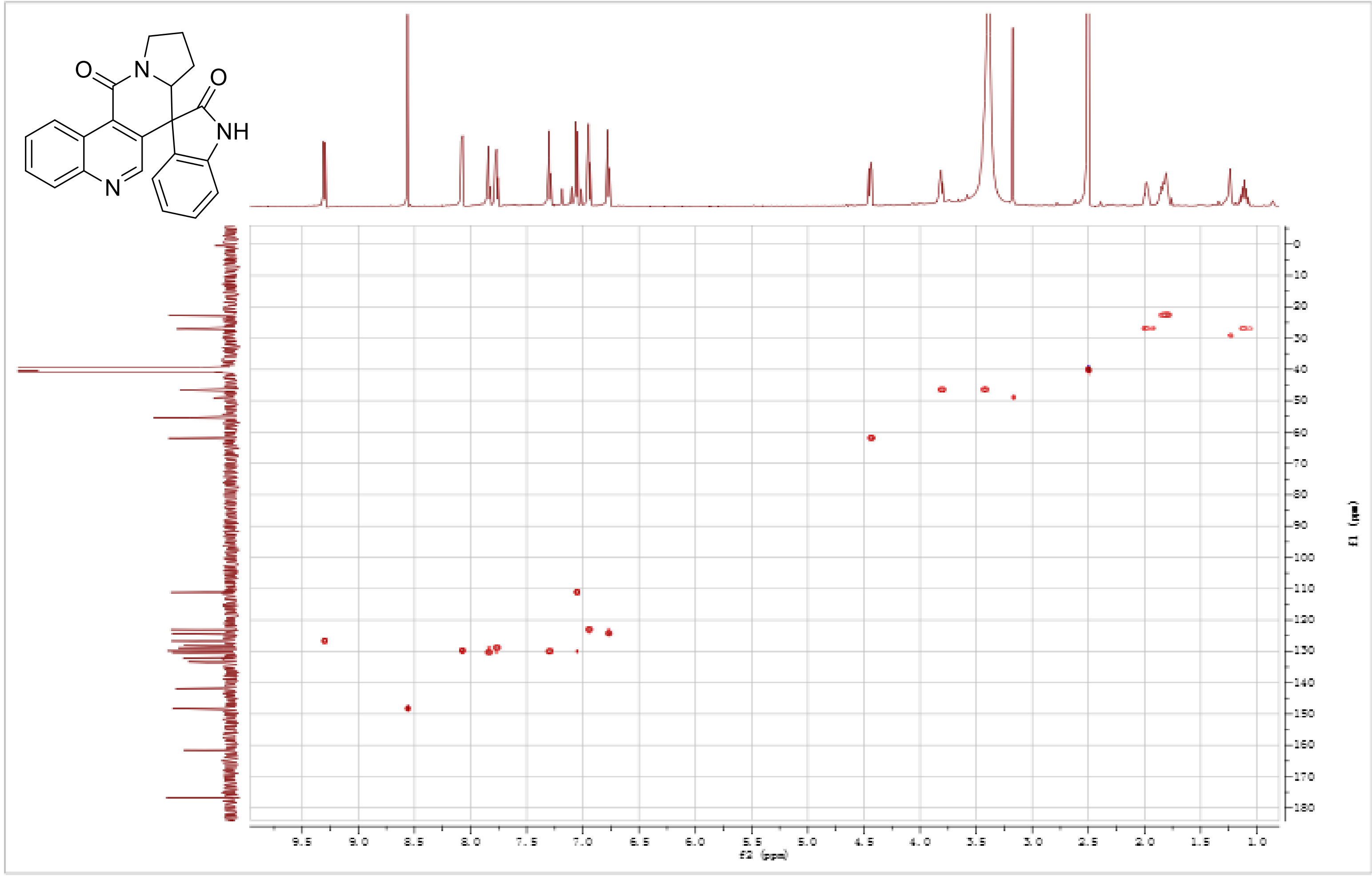

Figure S6. The HSQC spectrum of **1a/1b** (in DMSO-d_6)

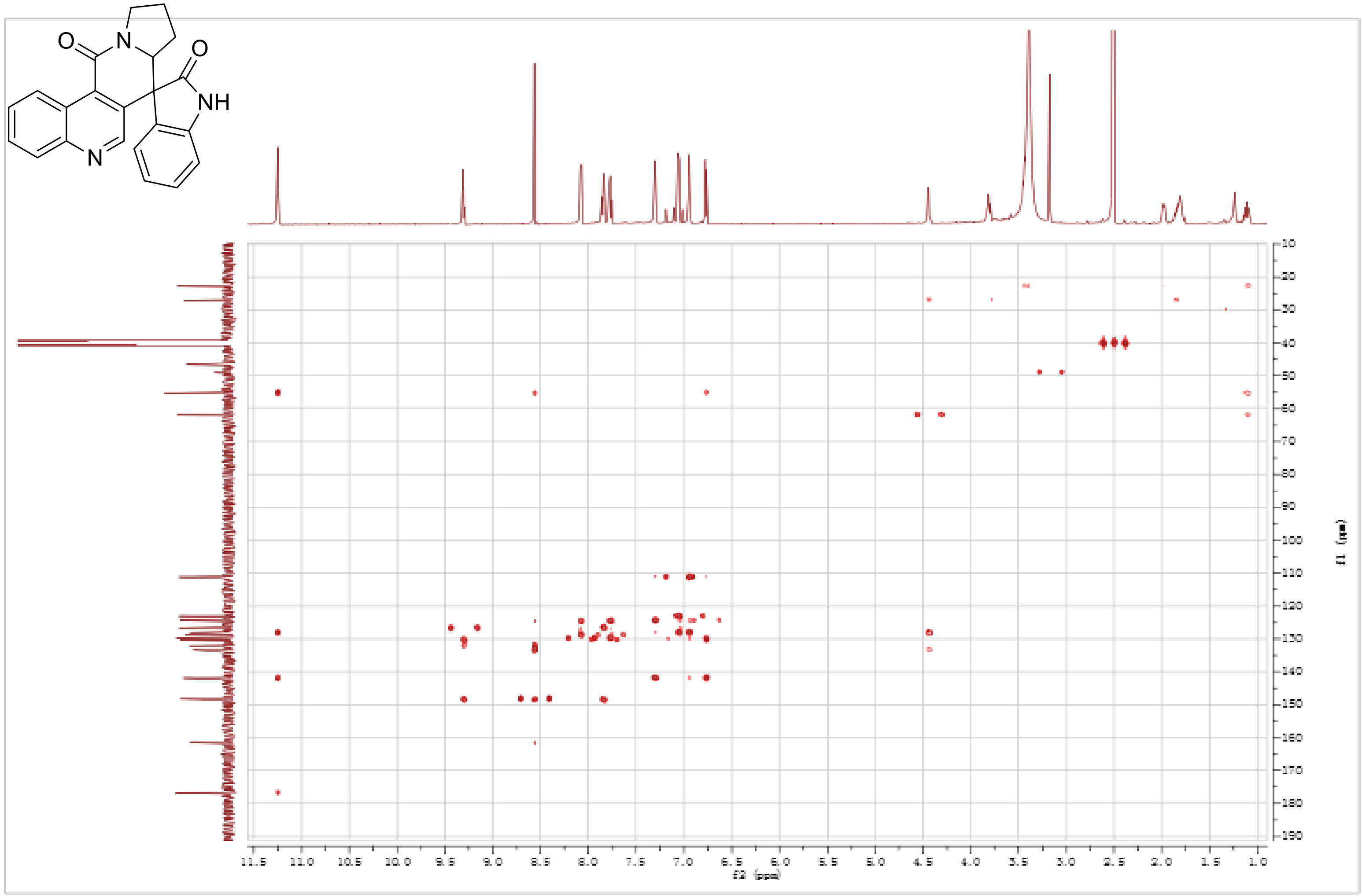

Figure S7. The HMBC spectrum of **1a/1b** (in DMSO-d_6)

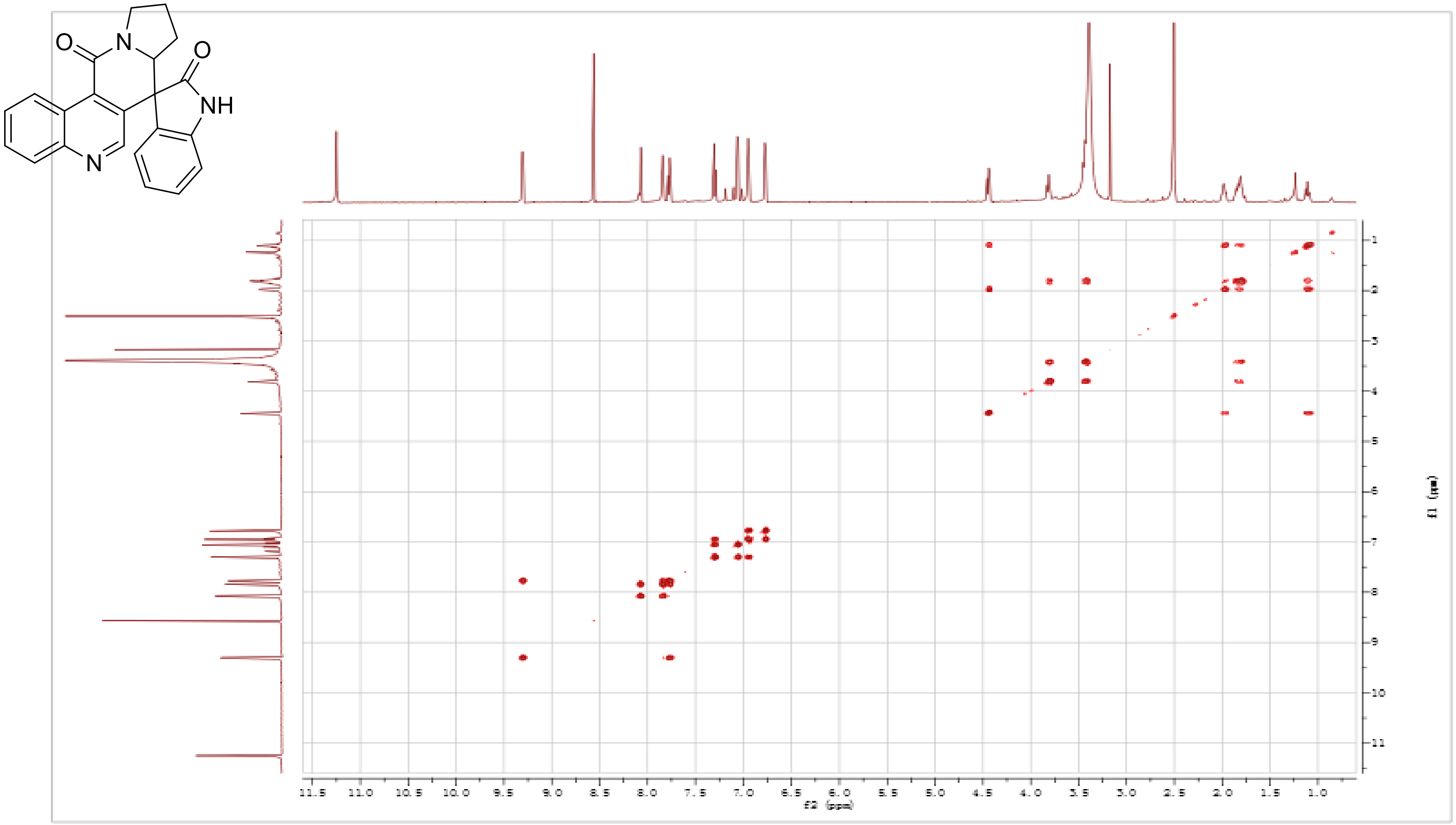

Figure S8. The ¹H-¹H COSY spectrum of **1a/1b** (in DMSO-d_6)

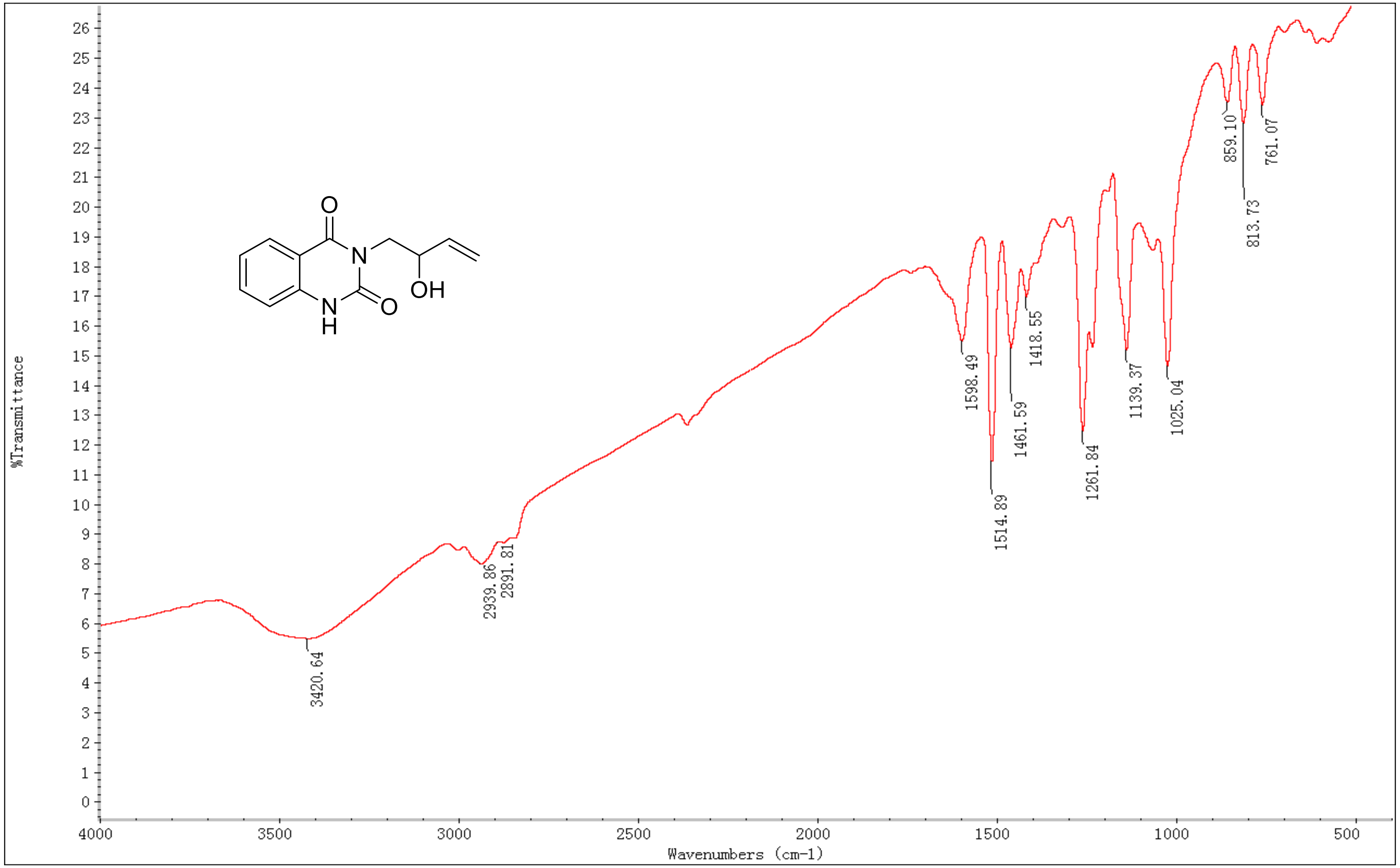

Figure S9. The IR spectrum of **2a/2b** (in KBr)

Qualitative Analysis Report

**Data Filename**	ESIH_20180917_ZWL_ZYT_43.d	**Sample Name**	ZYT-002-22
**Sample Type**	Sample	**Position**	P1-E4
**Instrument Name**	Agilent G6520 Q-TOF	**Acq Method**	20160324_MS_ESIH_NEG_1min.m
**Acquired Time**	9/17/2018 16:00:45	**IRM Calibration Status**	Success
**DA Method**	small molecular data analysis method.m	**Comment**	ESIH BY ZZY

User Spectra

Fragmentor Voltage	Collision Energy	Ionization Mode
175	0	ESI

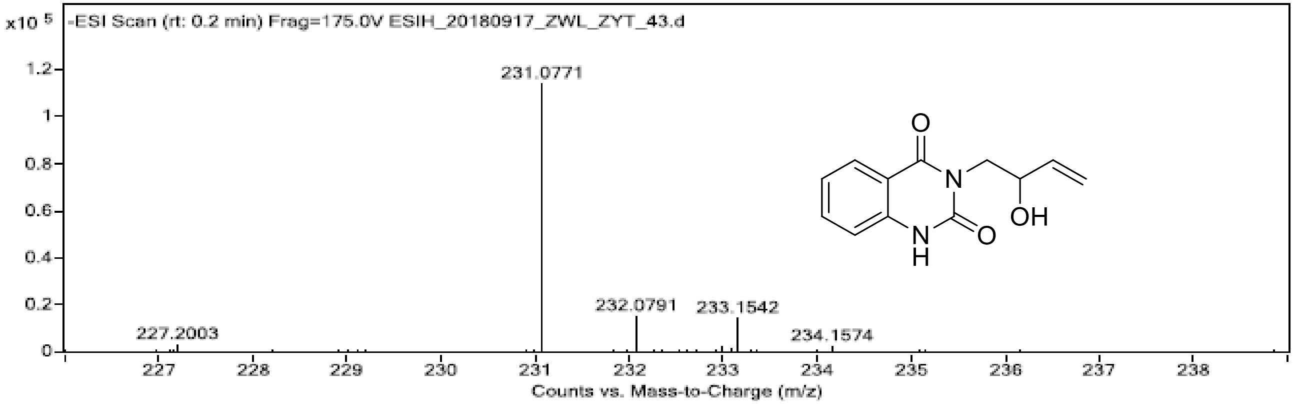

Formula Calculator Results

m/z	Calc m/z	Diff (mDa)	Diff (ppm)	Ion Formula	Ion
231.0771	231.0775	0.4	1.74	C12 H11 N2 O3	(M-H)-

--- End Of Report ---

Figure S10. The HR-ESI-MS spectrum of **2a/2b** (in MeOH)

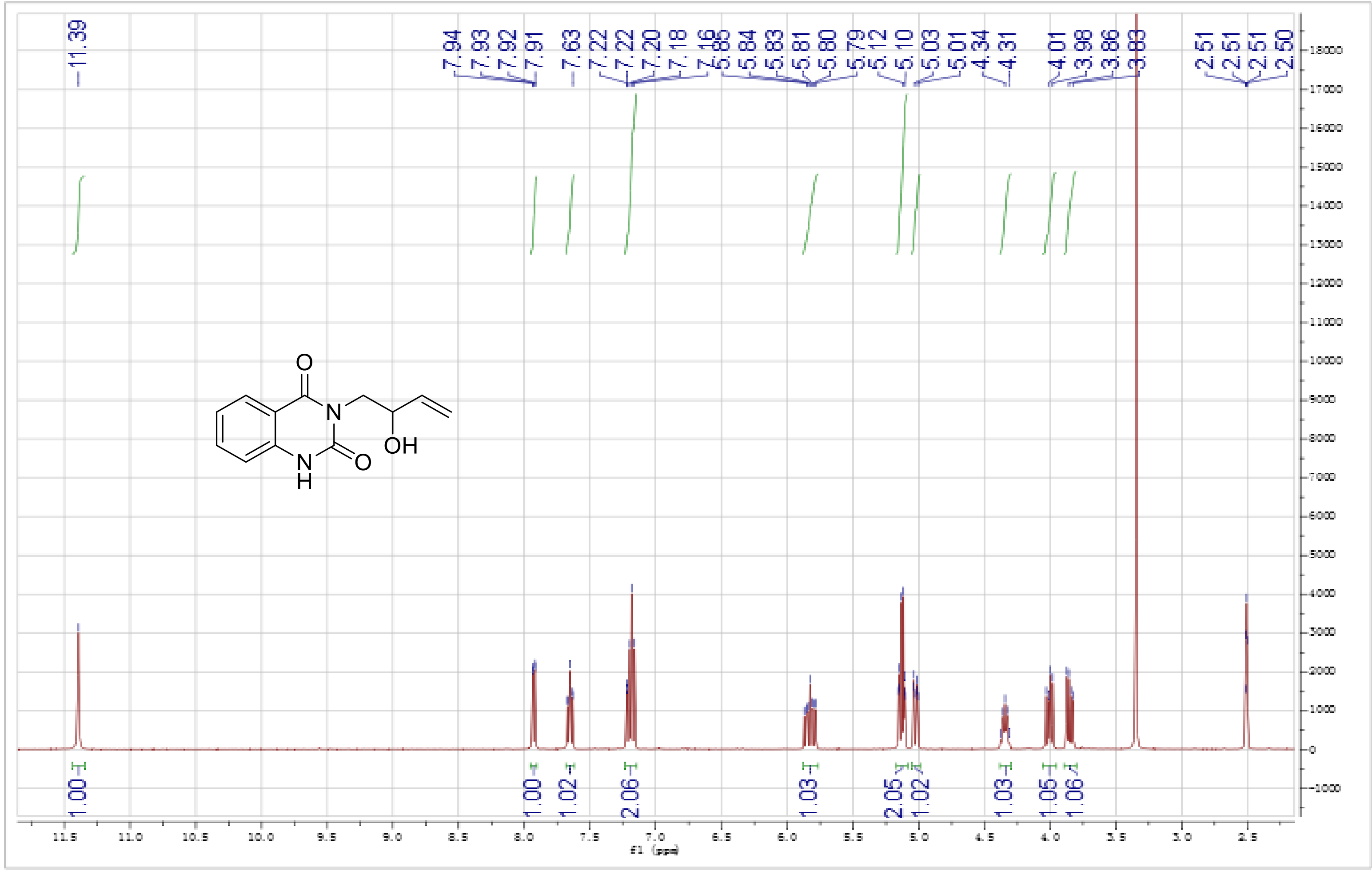

Figure S11. The ^{1}H NMR spectrum of **2a/2b** (in DMSO-d_6)

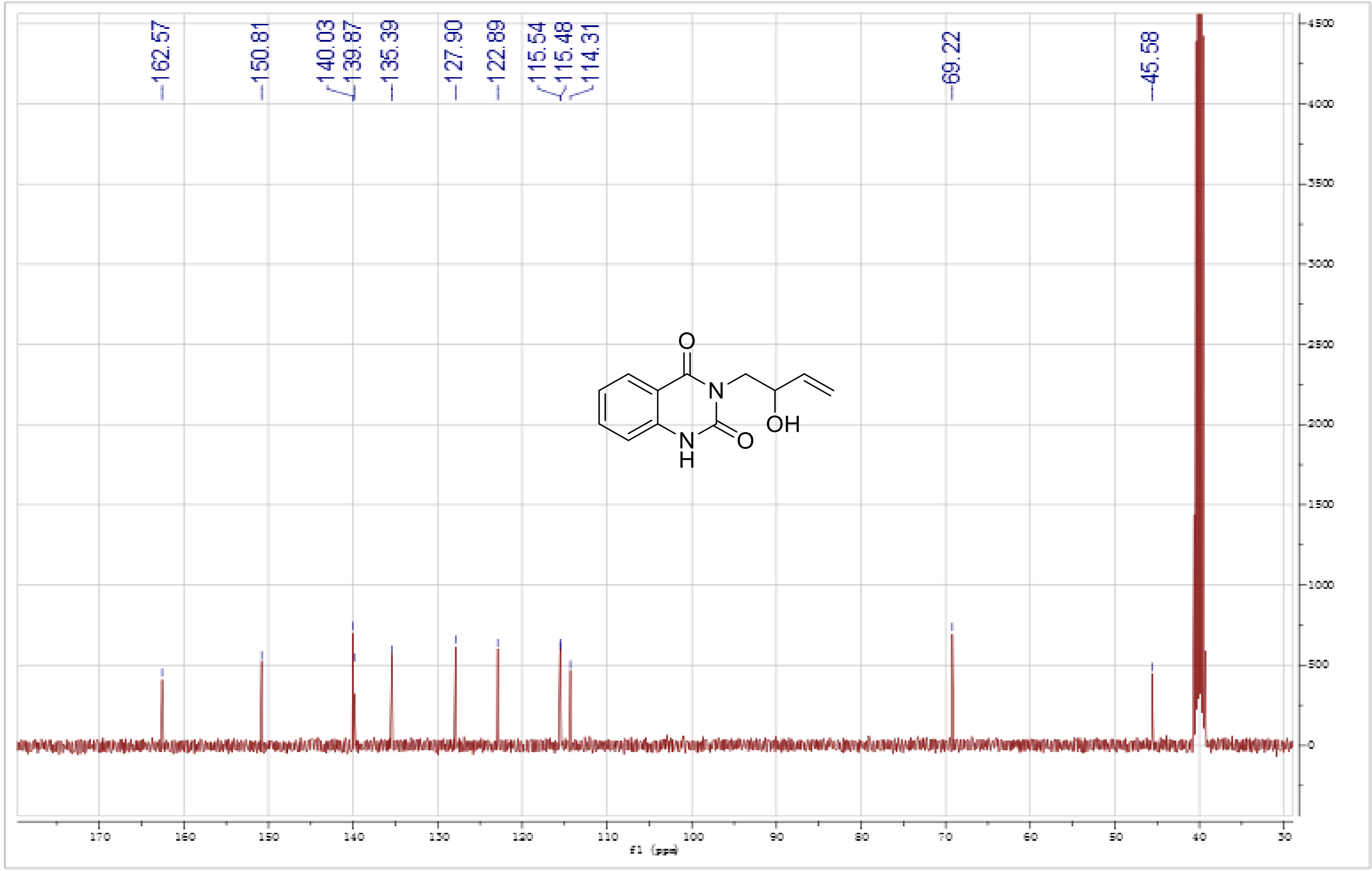

Figure S12. The ^{13}C NMR spectrum of **2a/2b** (in DMSO-d_6)

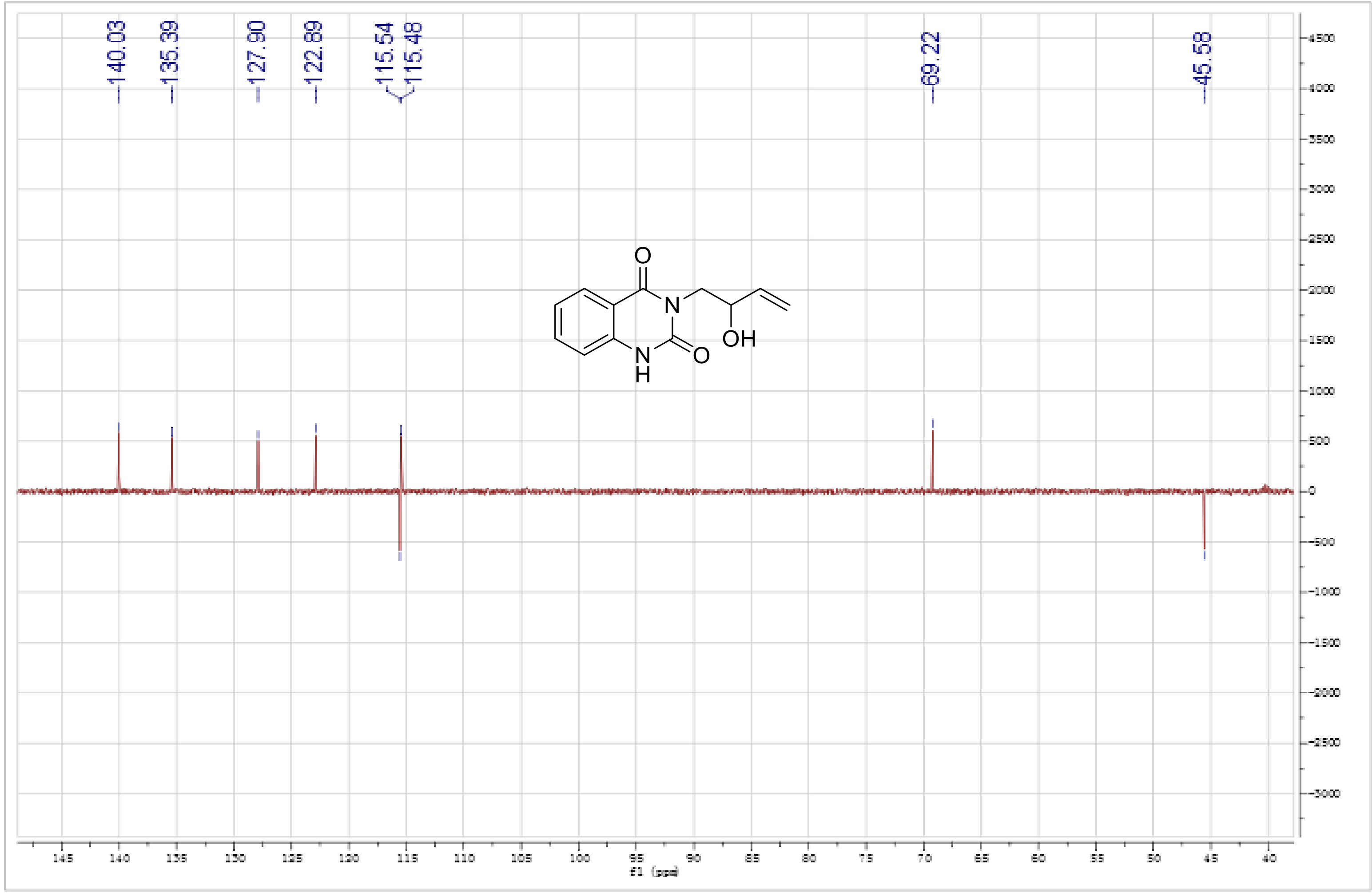

Figure S13. The DEPT 135° spectrum of **2a/2b** (in DMSO-d_6)

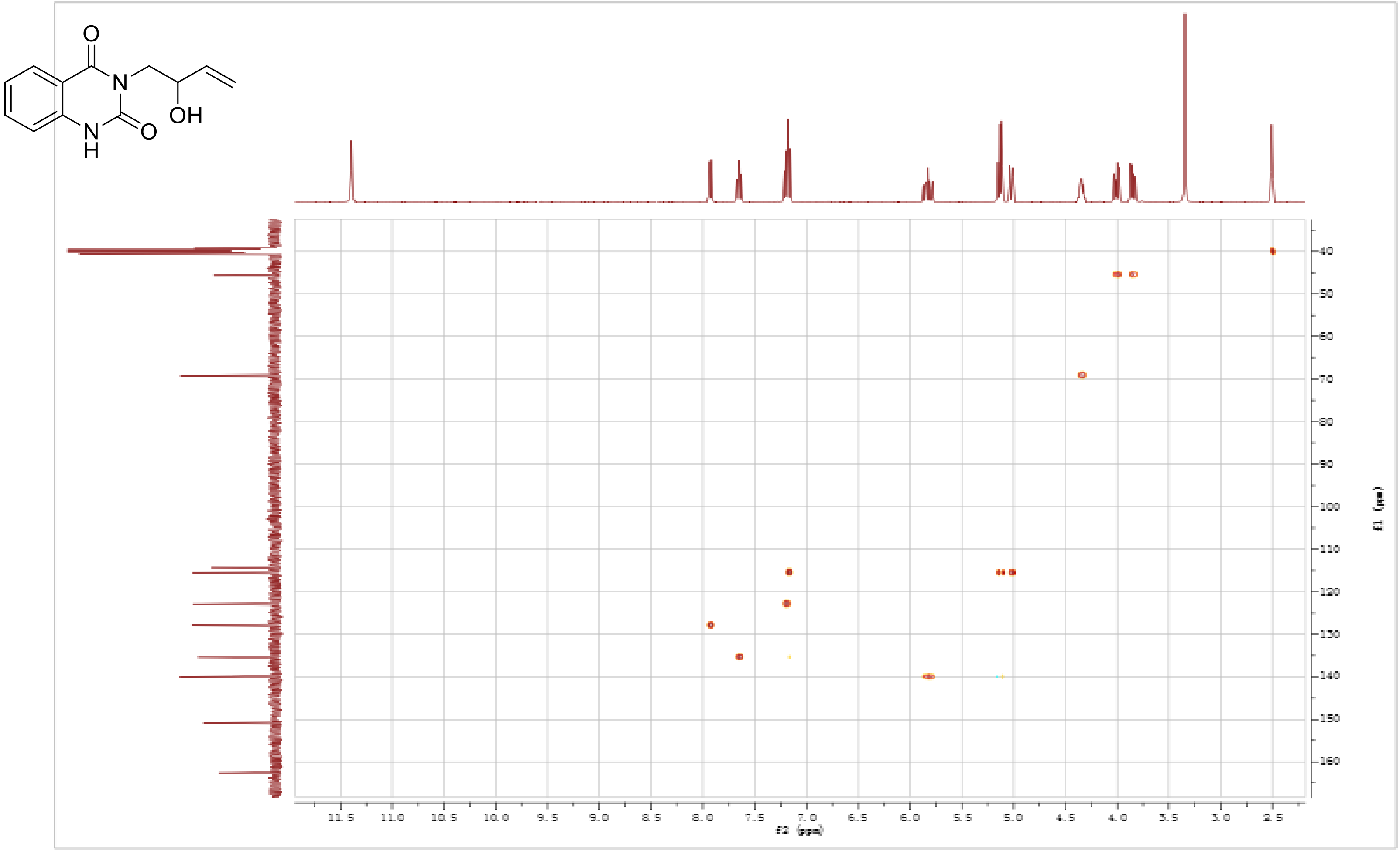

Figure S14. The HSQC spectrum of **2a/2b** (in DMSO-d_6)

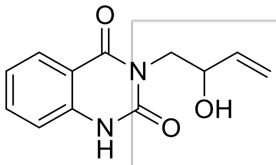

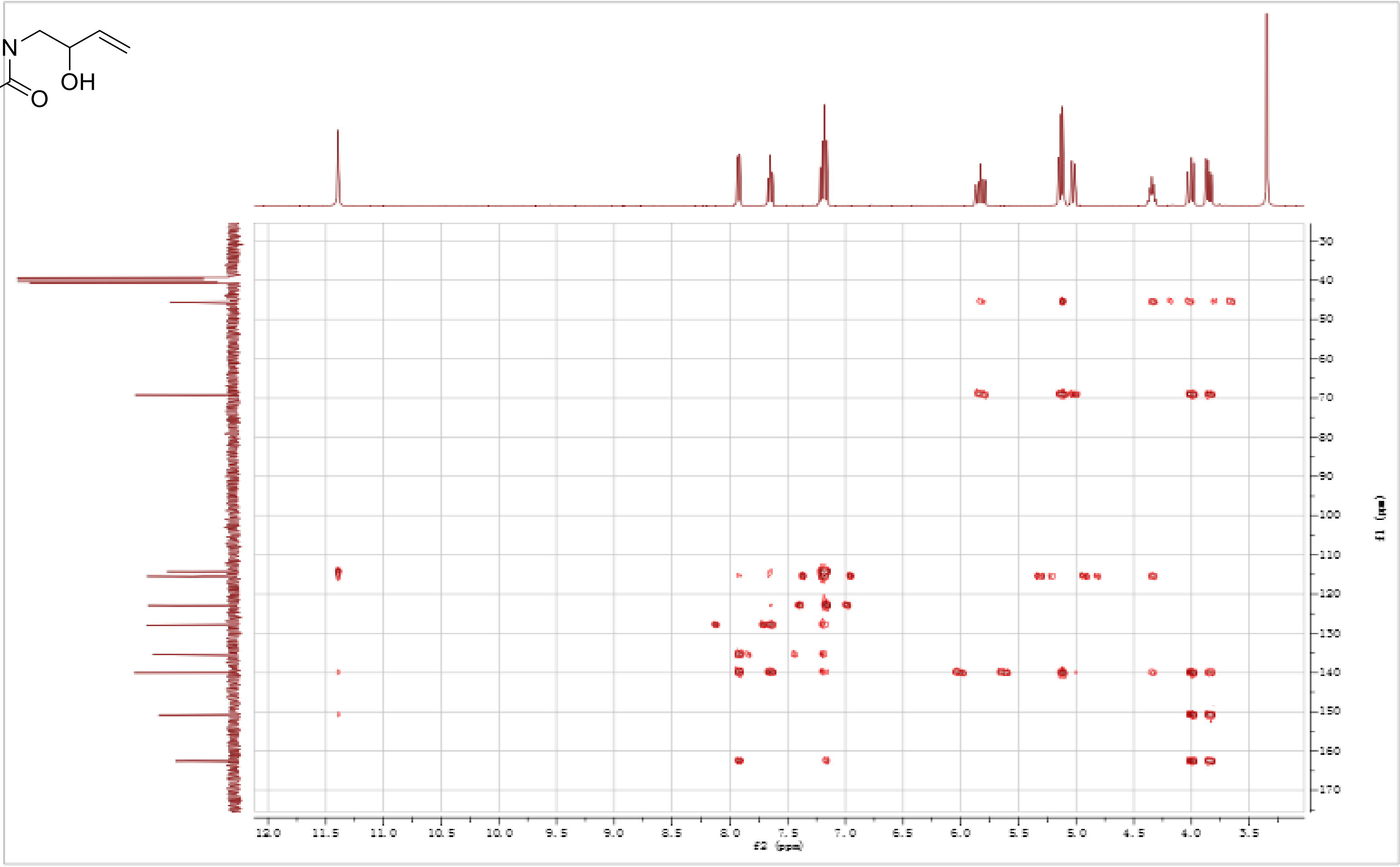

Figure S15. The HMBC spectrum of **2a/2b** (in DMSO-d_6)

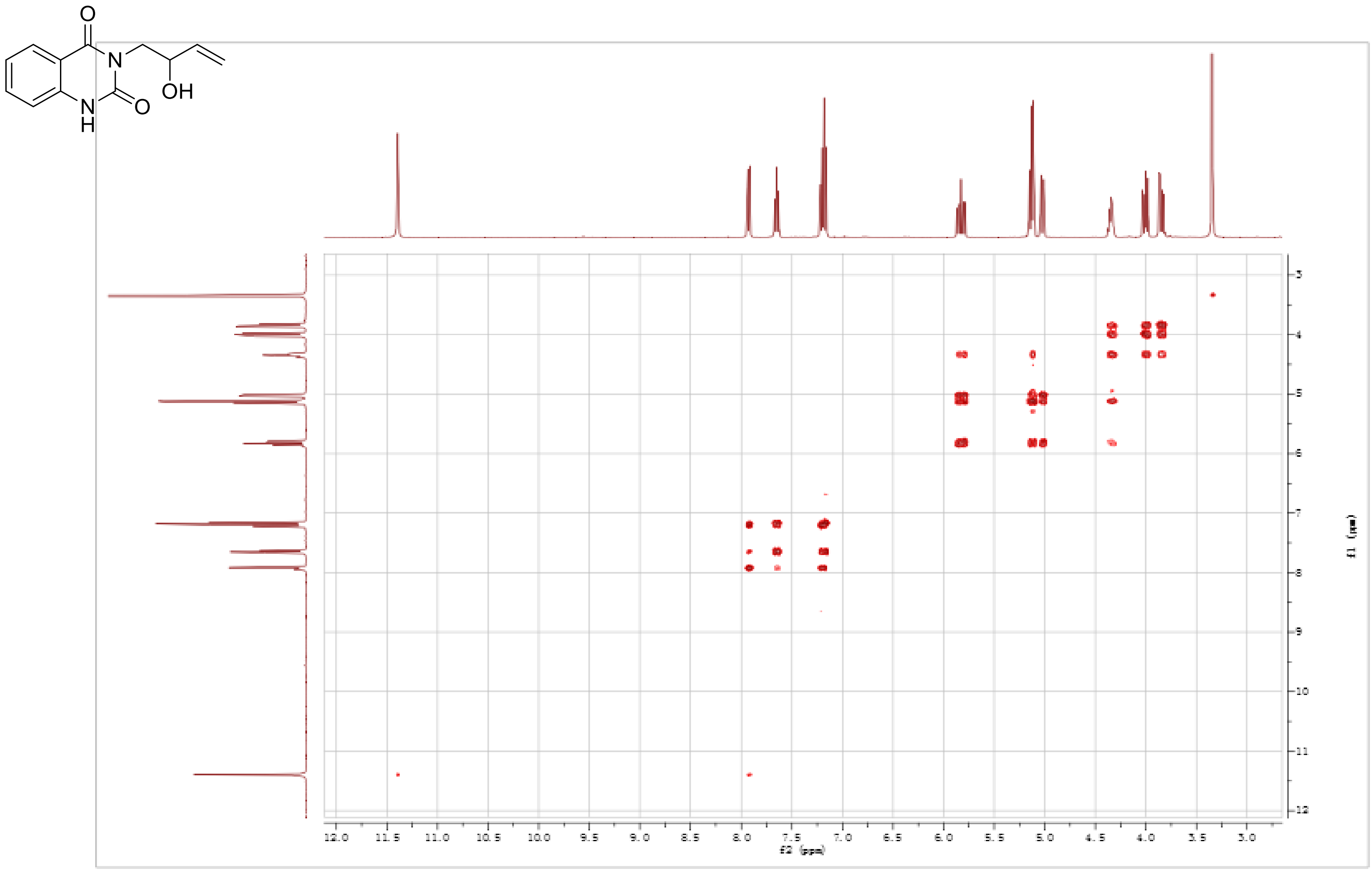

Figure S16. The ^{1}H-^{1}H COSY spectrum of **2a/2b** (in DMSO-d_6)

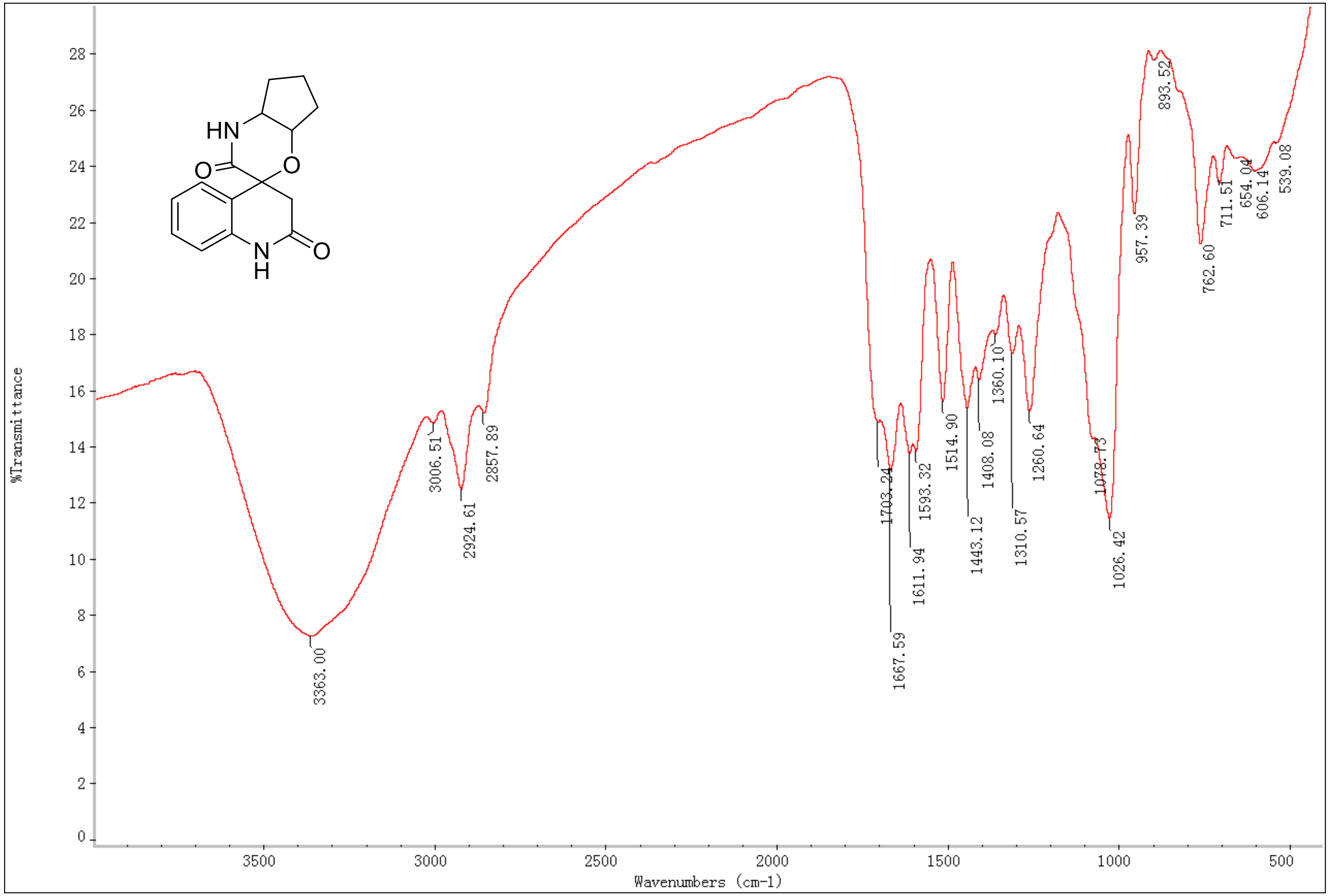

Figure S17. The IR spectrum of **3a/3b** (in KBr)

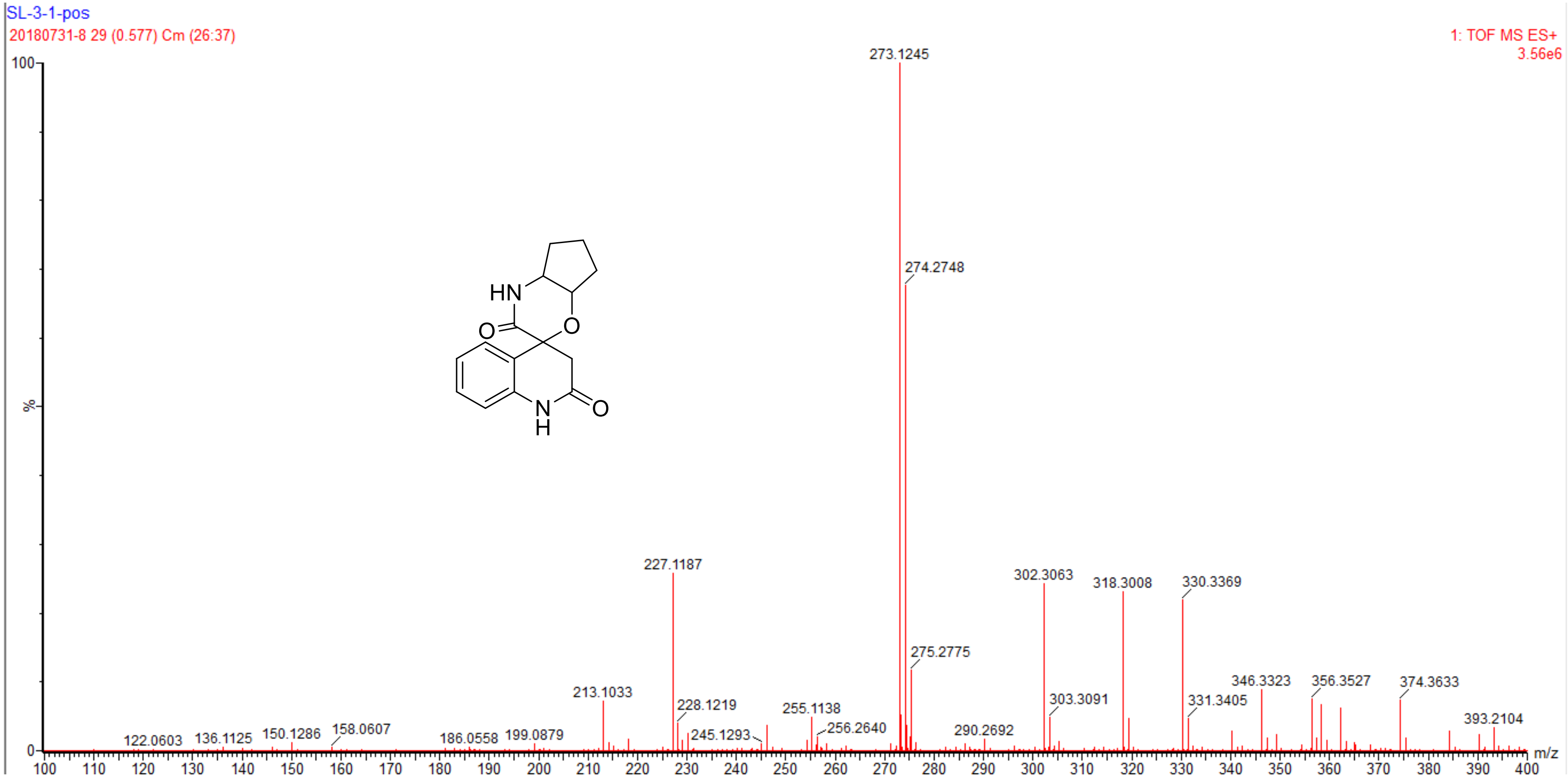

Figure S18. The HR-ESI-MS spectrum of **3a/3b** (in MeOH)

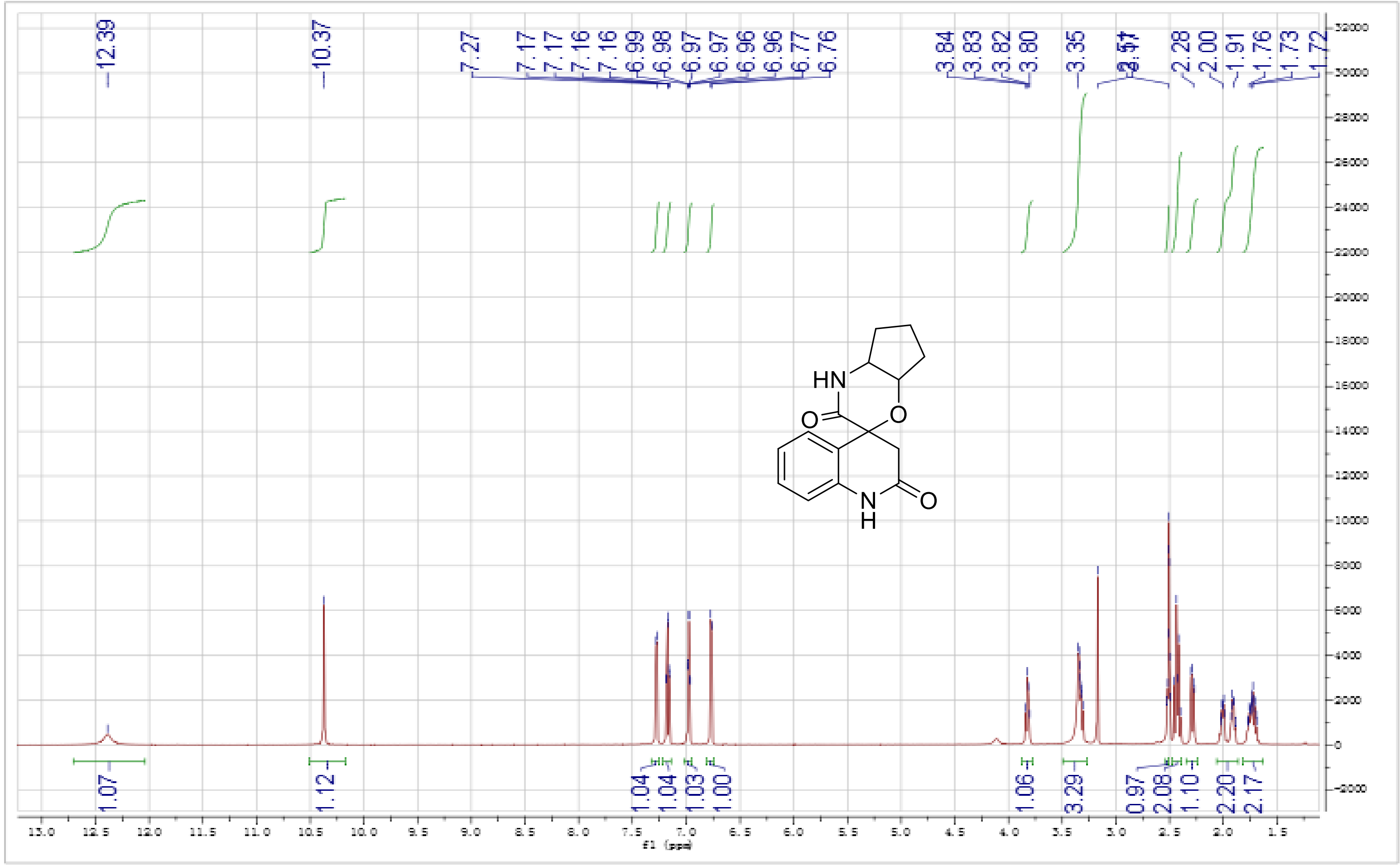

Figure S19. The ^{1}H NMR spectrum of **3a/3b** (in DMSO-d_6)

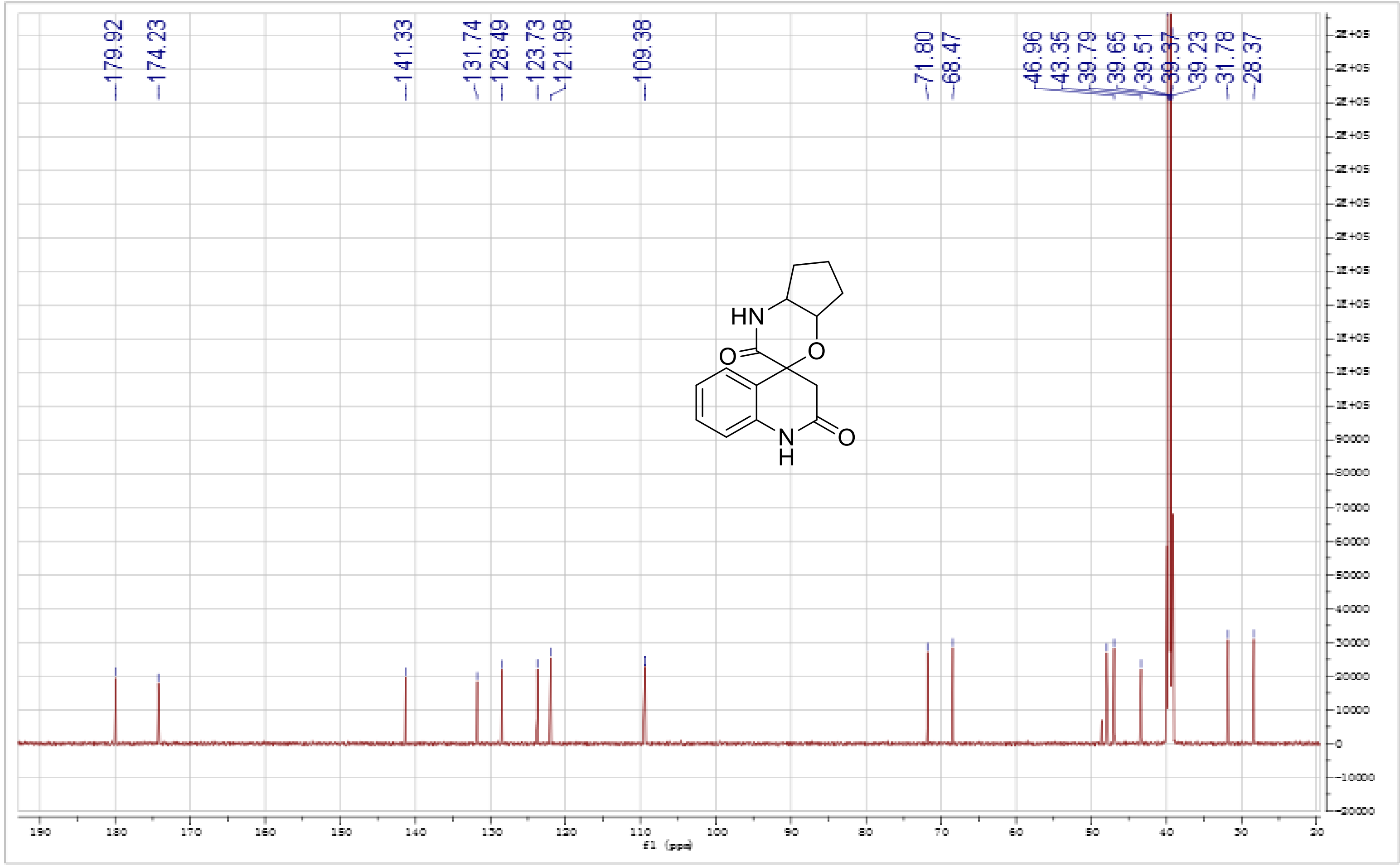

Figure S20. The ^{13}C NMR spectrum of **3a/3b** (in DMSO-d_6)

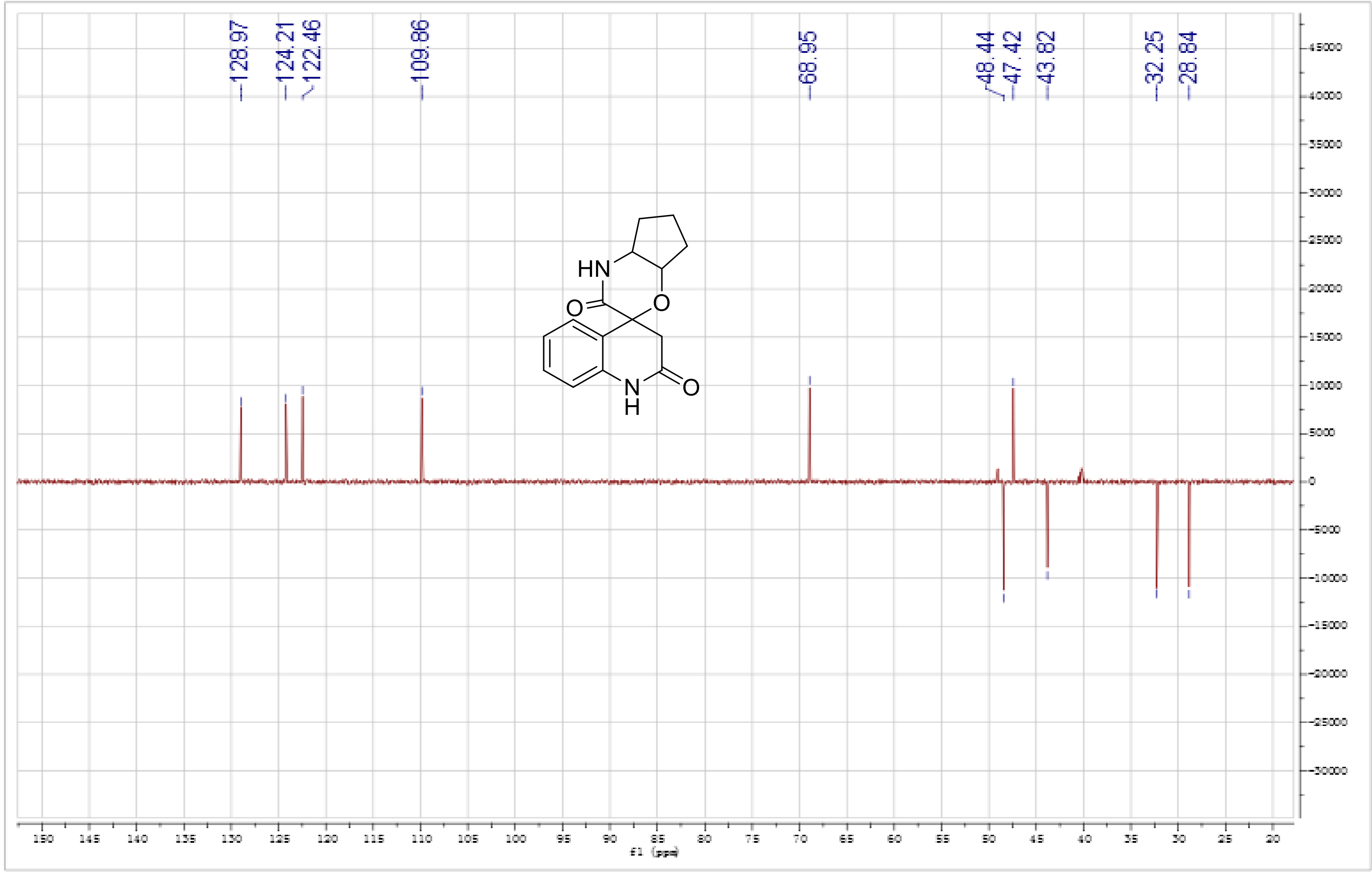

Figure S21. The DEPT 135° spectrum of **3a**/**3b** (in DMSO-d_6)

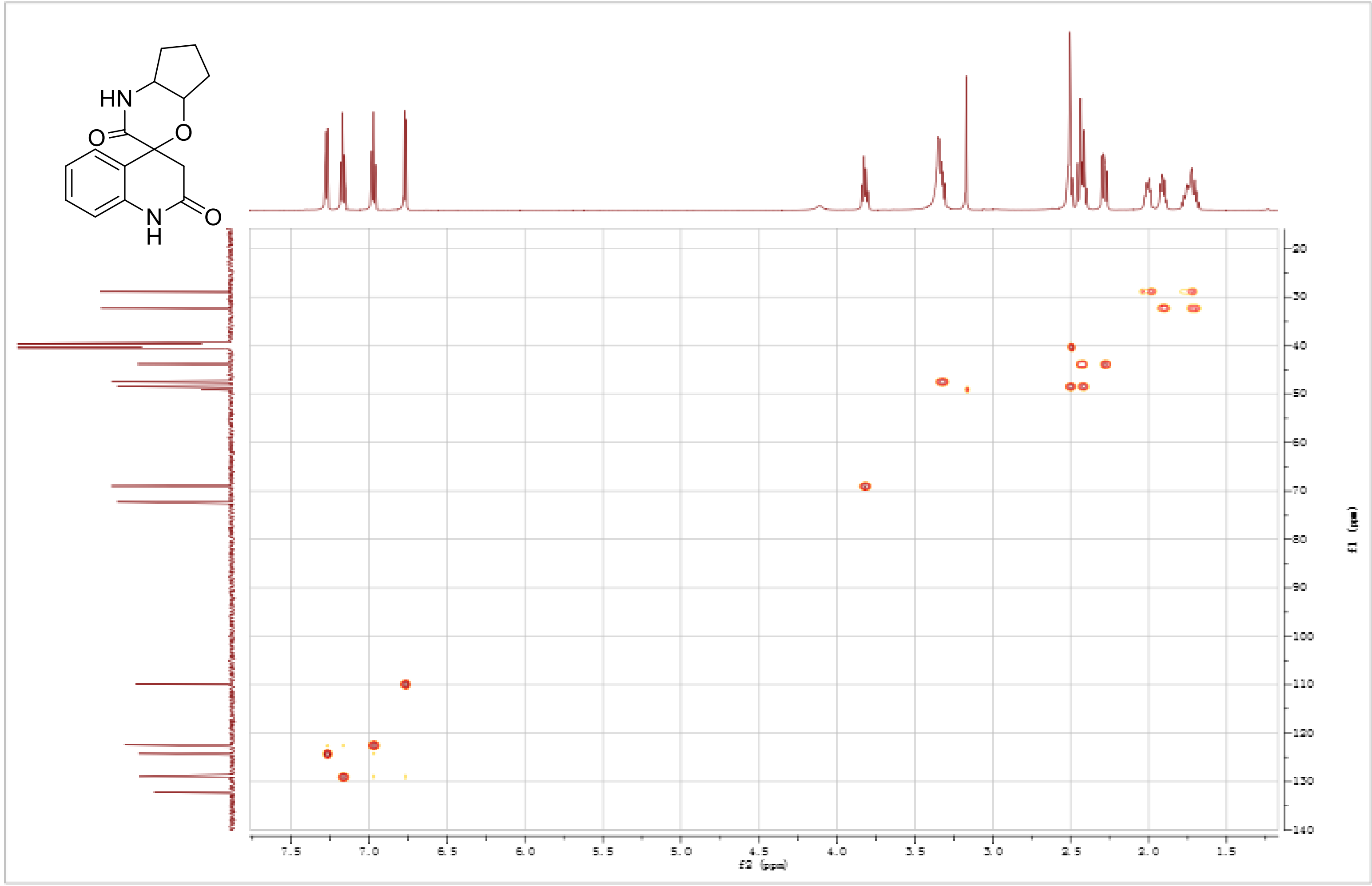

Figure S22. The HSQC spectrum of **3a/3b** (in DMSO-d_6)

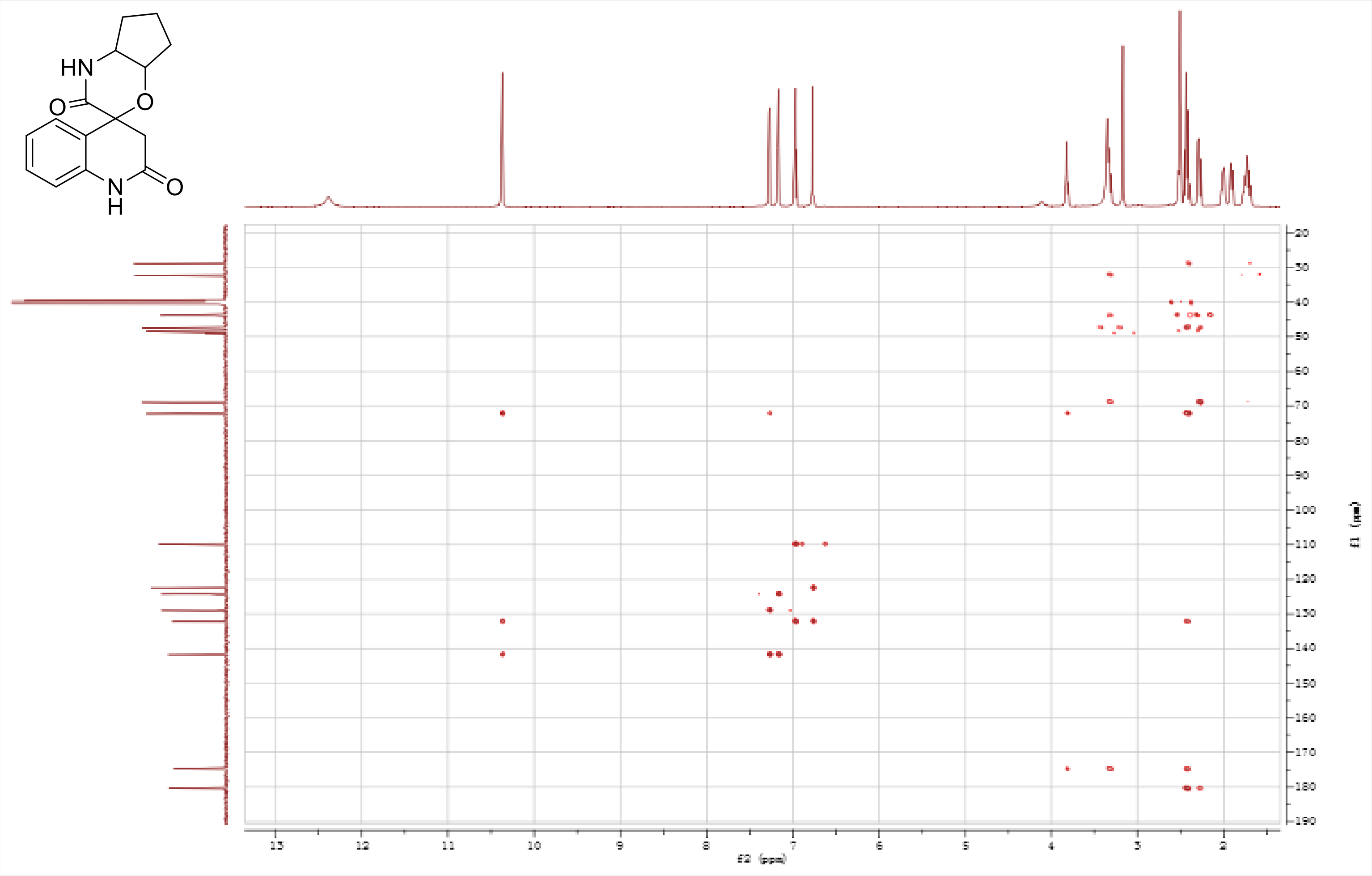

Figure S23. The HMBC spectrum of **3a/3b** (in DMSO-d_6)

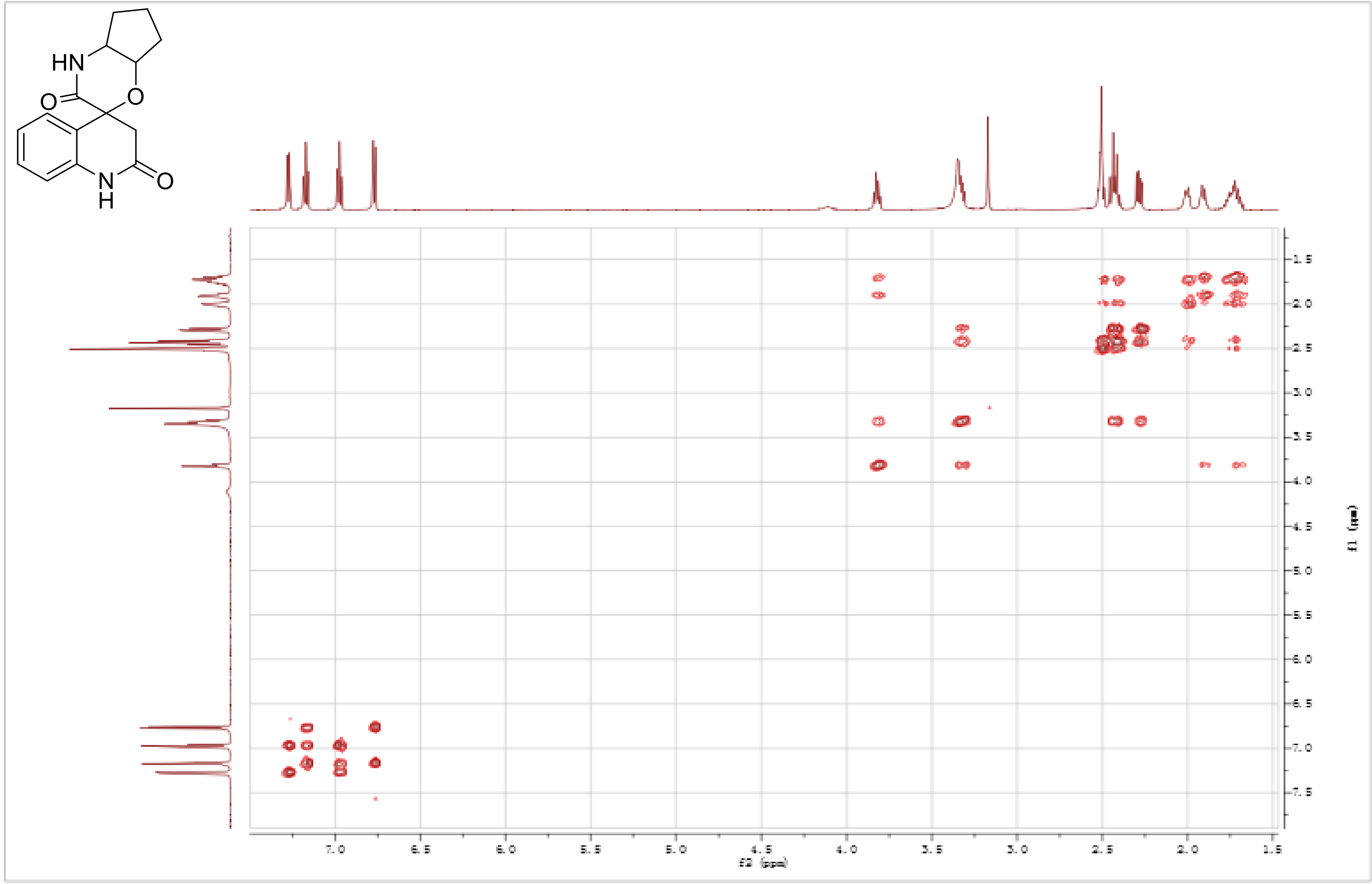

Figure S24. The ¹H-¹H COSY spectrum of **3a/3b** (in DMSO-d_6)

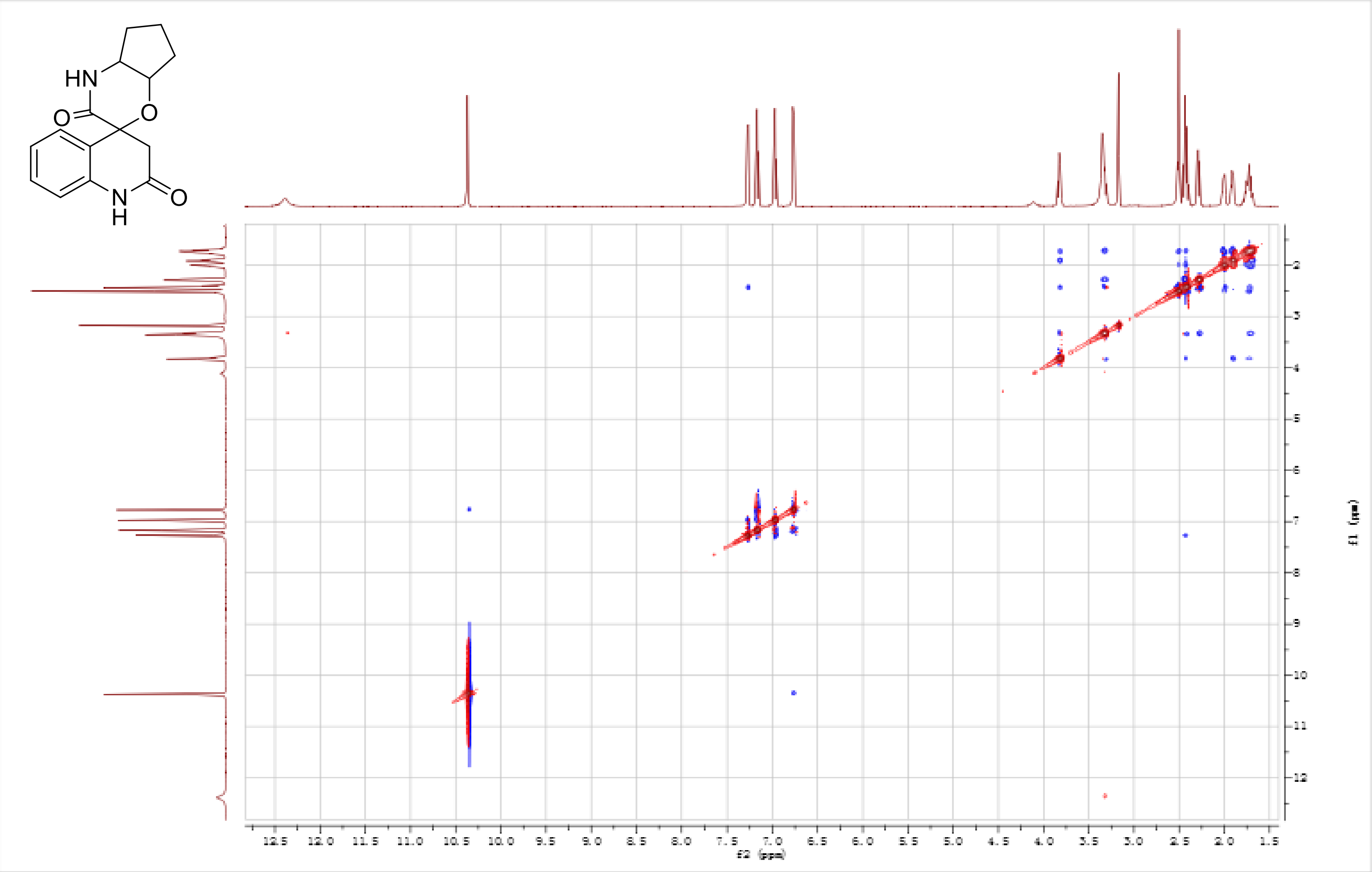

Figure S25. The ROESY spectrum of **3a/3b** (in DMSO-d_6)

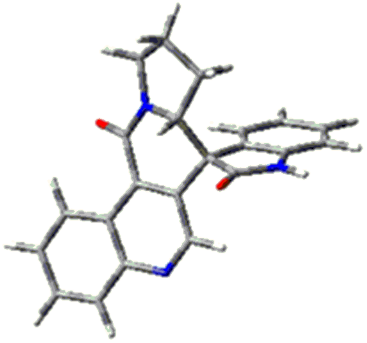

(3'*S*,1"*R*)-**1**-1
90.05%

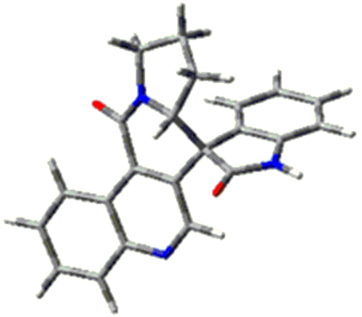

(3'*S*,1"*R*)-**1**-2
9.95%

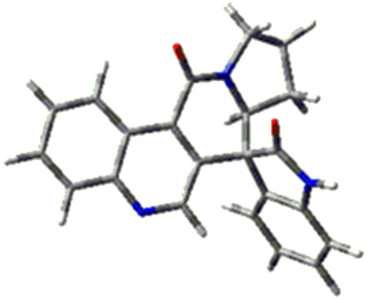

(3'*S*,1"*S*)-**1**-1
91.10%

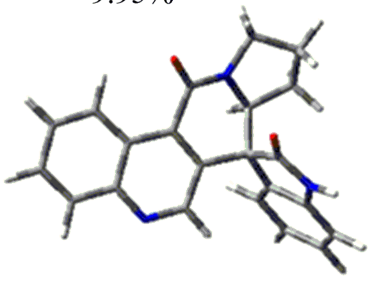

(3'*S*,1"*S*)-**1**-2
8.90%

Figure S26. b3lyp/6-31g(d) optimized lowest energy conformers for (3'*S*,2"*R*)-**1** and (3'*S*,2"*S*)-**1** and their equilibrium populations

The experimental ECD spectrum of **1a** (red line) and **1b** (blue line) and the calculated ECD spectrum of (3'*S*,2"*R*)-**1** (red short dash), (3'*R*,2"*S*)-**1** (blue short dash), (3'*S*,2"*S*)-**1** (green short dash) and (3'*R*,2"*R*)-**1** (light blue short dash). The calculated ECD (excited states 30) spectrum were plotted as sums of Gaussians 09 with a 0.22 eV exponential half-width using the program Specdis 1.62, and the UV shifted was 3 nm.

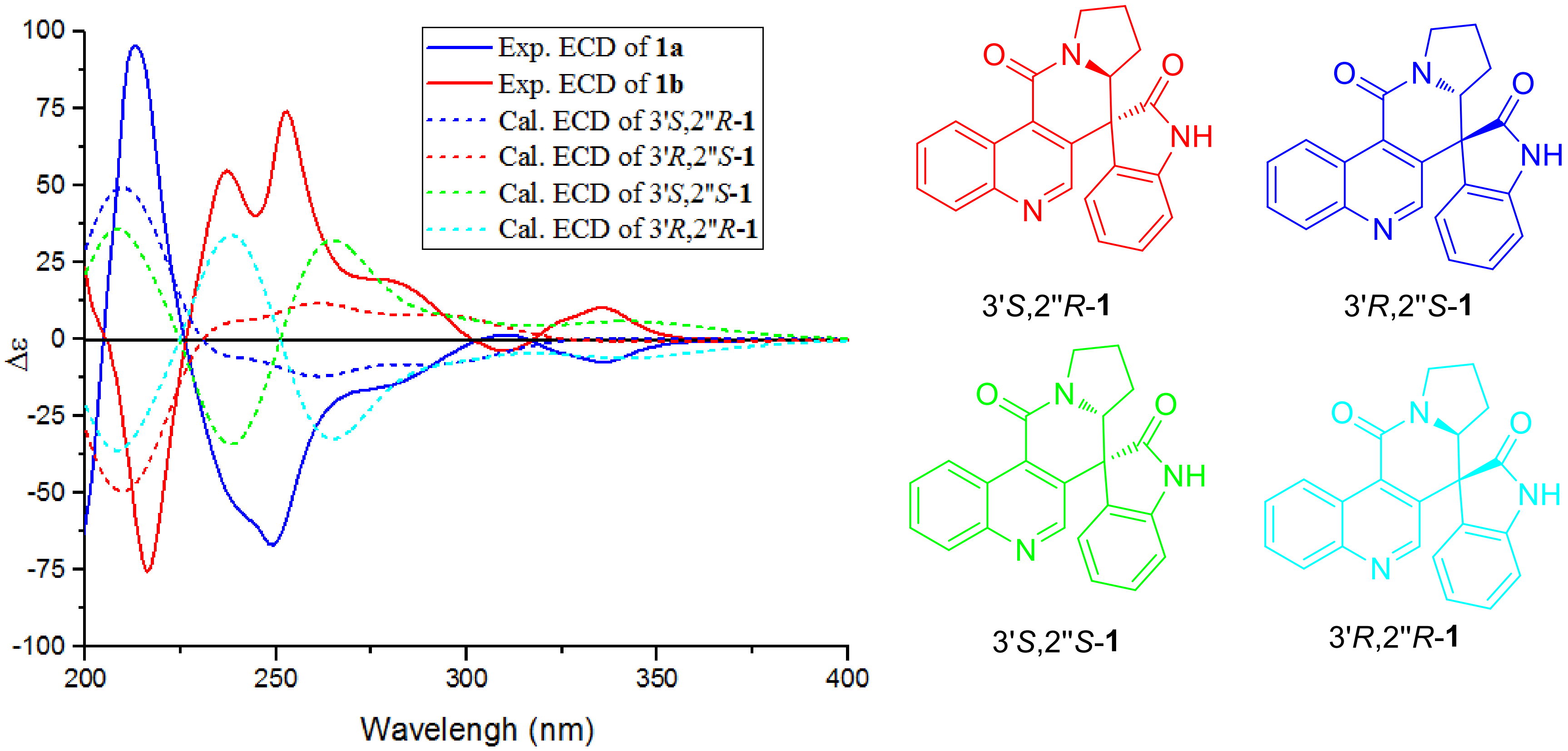

Figure S27. Experimental and calculated ECD spectrum of **1**

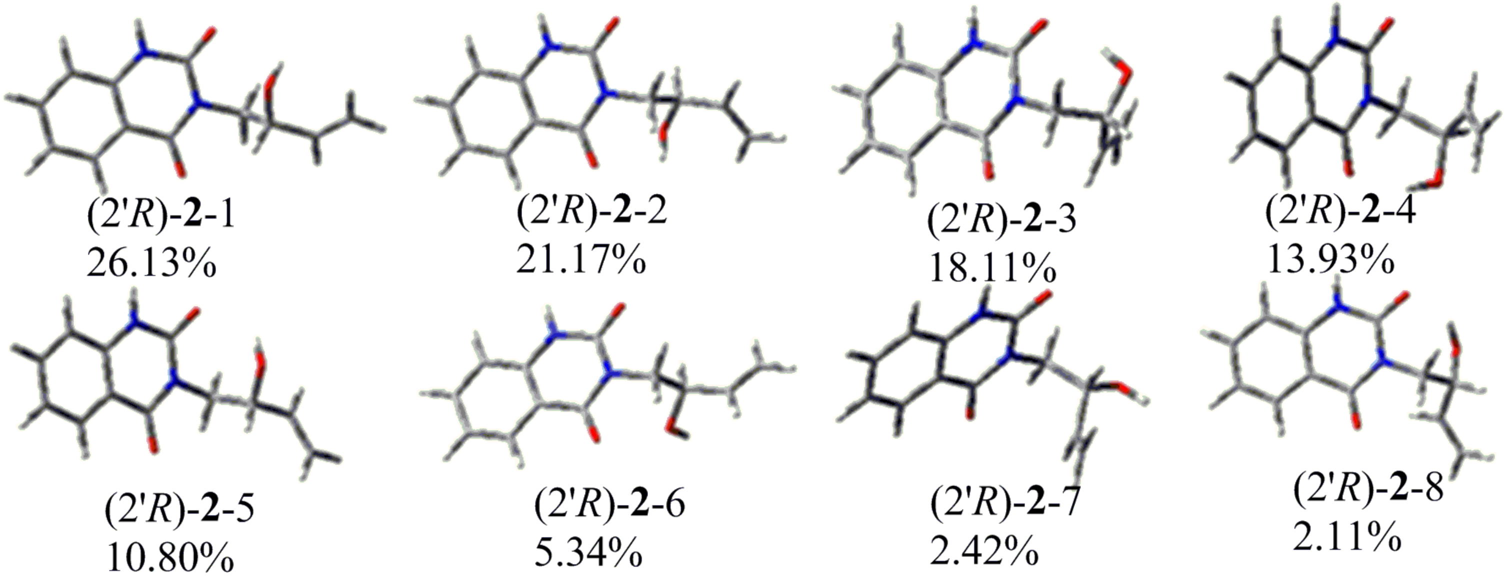

Figure S28. b3lyp/6-31g(d) optimized lowest energy conformers for (2'*R*)-**2** and their equilibrium populations

The experimental ECD spectrum of **2a** (red line) and **2b** (black line) and the calculated ECD spectrum of (2'*R*)-**2** (red short dash) and (2'*S*)-**2** (black short dash). The calculated ECD (excited states 30) spectrum were plotted as sums of Gaussians 09 with a 0.16 eV exponential half-width using the program Specdis 1.62, and the UV shifted was7 nm.

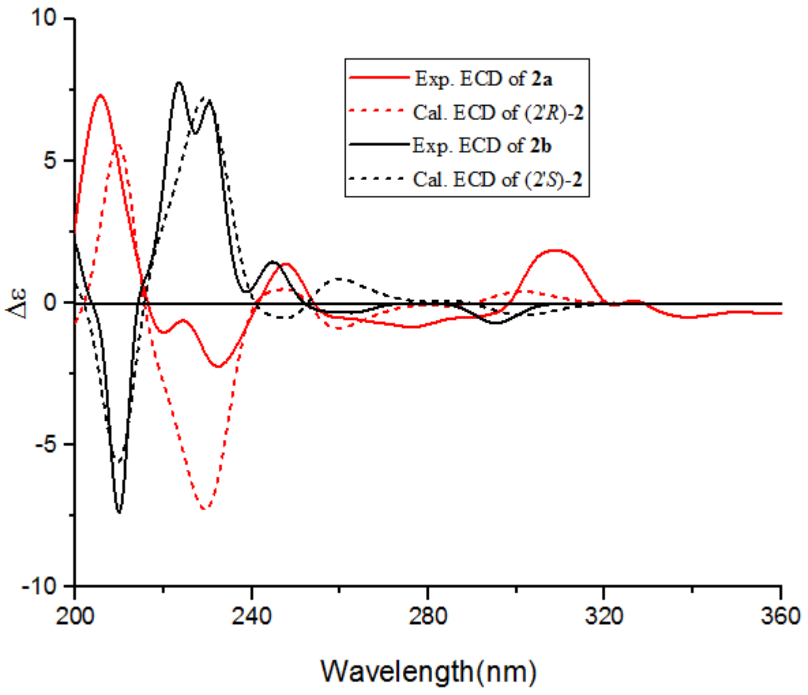

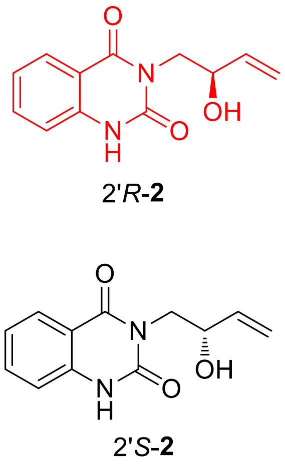

Figure S29. Experimental and calculated ECD spectrum of **2**

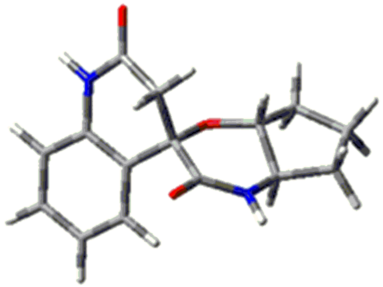

(4*S*,2'*R*,3'*R*)-**3**-1
62.62%

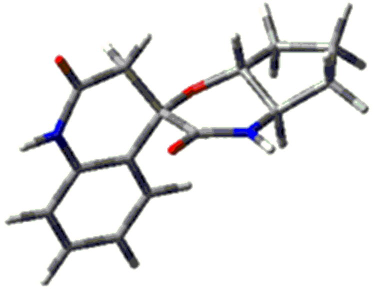

(4*S*,2'*R*,3'*R*)-**3**-2
37.38%

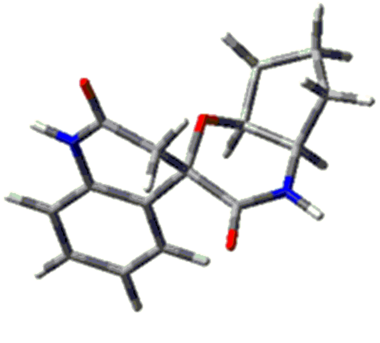

(4*S*,2'*S*,3'*R*)-**3**-1
41.69%

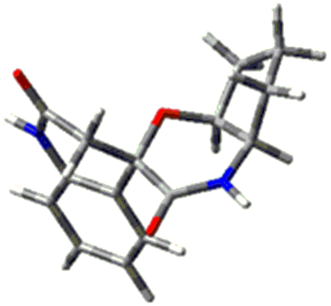

(4*S*,2'*S*,3'*R*)-**3**-2
41.63%

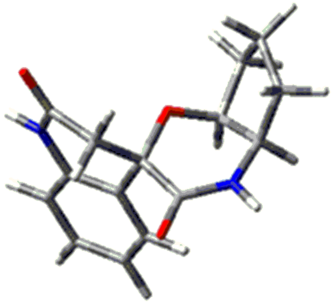

(4*S*,2'*S*,3'*R*)-**3**-3
16.69%

Figure S30. b3lyp/6-31g(d) optimized lowest energy conformers for (4*S*,2'*R*,3'*R*)-**3** and (4*S*,2'*S*,3'*R*)-**3** and their equilibrium populations

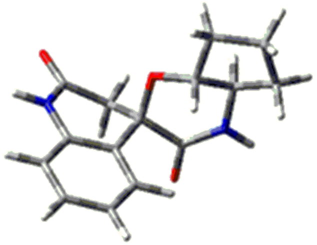

(4*R*,2'*R*,3'*R*)-**3**-1
95.59%

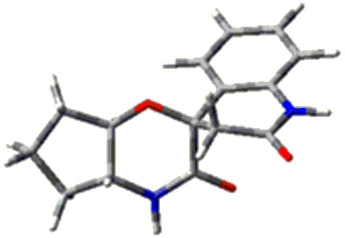

(4*R*,2'*R*,3'*R*)-**3**-2
4.05%

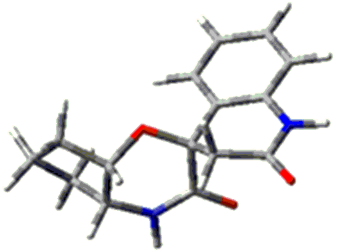

(4*R*,2'*S*,3'*R*)-**3**-1
17.72%

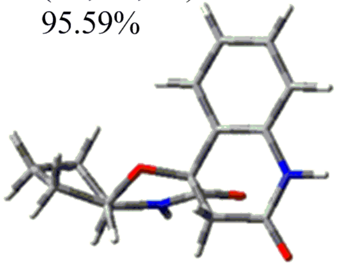

(4*R*,2'*S*,3'*R*)-**3**-2
17.71%

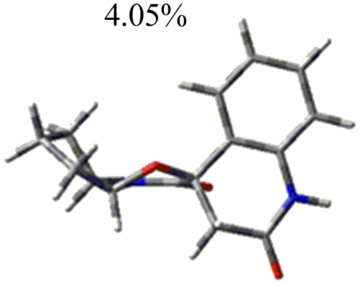

(4*R*,2'*S*,3'*R*)-**3**-3
17.71%

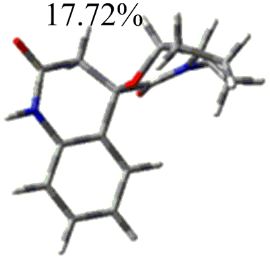

(4*R*,2'*S*,3'*R*)-**3**-4
15.62%

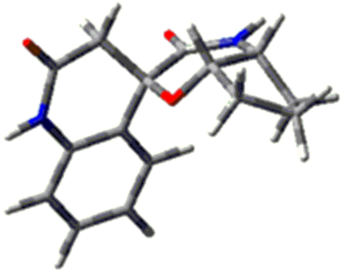

(4*R*,2'*S*,3'*R*)-**3**-5
15.61%

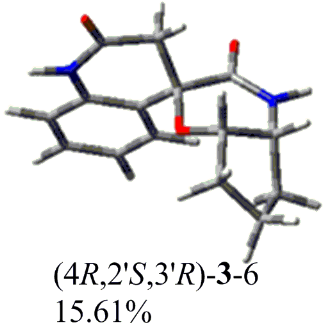

(4*R*,2'*S*,3'*R*)-**3**-6
15.61%

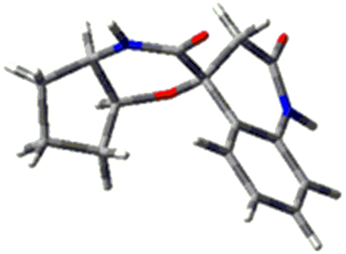

(4*R*,2'*S*,3'*R*)-**3**-7
0.03%

Figure S31. b3lyp/6-31g(d) optimized lowest energy conformers for (4*R*,2'*R*,3'*R*)-**3** and (4*R*,2'*S*,3'*R*)-**3** and their equilibrium populations

The experimental ECD spectrum of **3a** (red line) and **3b** (blue line) and the calculated ECD spectrum of (4*S*,2'*R*,3'*R*)-**3** (red short dash), (4*R*,2'*S*,3'*S*)-**3** (blue short dash), (4*S*,2'*S*,3'*R*)-**3** (green short dash) and (4*R*,2'*R*,3'*S*)-**3** (light blue short dash). The calculated ECD (excited states 30) spectrum were plotted as sums of Gaussians 09 with a 0.28 eV exponential half-width using the program Specdis 1.62, and the UV shifted was 2 nm.

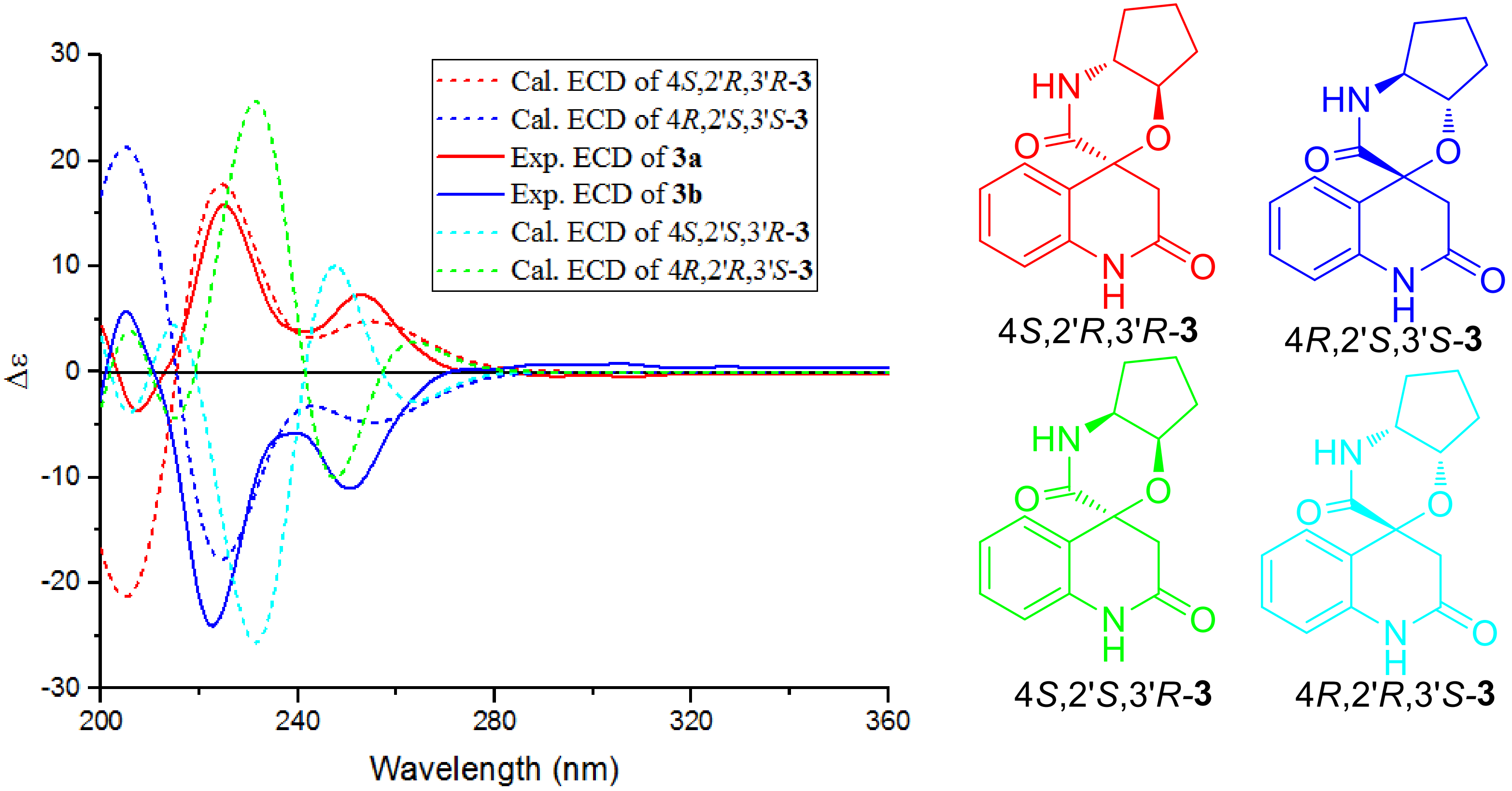

Figure S32. Experimental and calculated ECD spectrum of **3**

The experimental ECD spectrum of **3a** (red line) and **3b** (blue line) and the calculated ECD spectrum of (4*R*,2'*R*,3'*R*)-**3** (red short dash), (4*S*,2'*S*,3'*S*)-**3** (blue short dash), (4*R*,2'*S*,3'*R*)-**3** (green short dash) and (4*S*,2'*R*,3'*S*)-**3** (light blue short dash). The calculated ECD (excited states 30) spectrum were plotted as sums of Gaussians 09 with a 0.28 eV exponential half-width using the program Specdis 1.62, and the UV shifted was 2 nm.

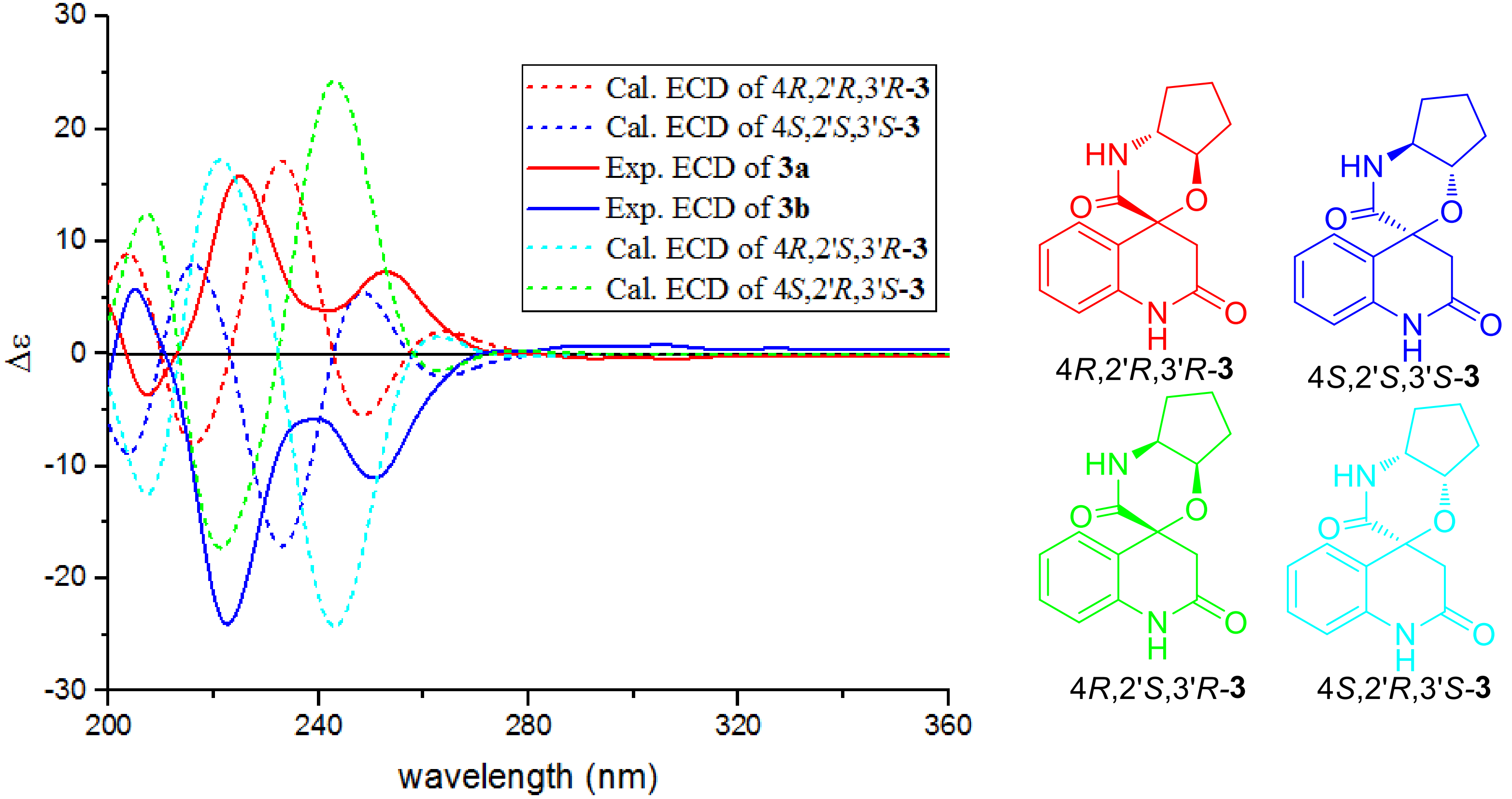

Figure S33. Experimental and calculated ECD spectrum of **3**

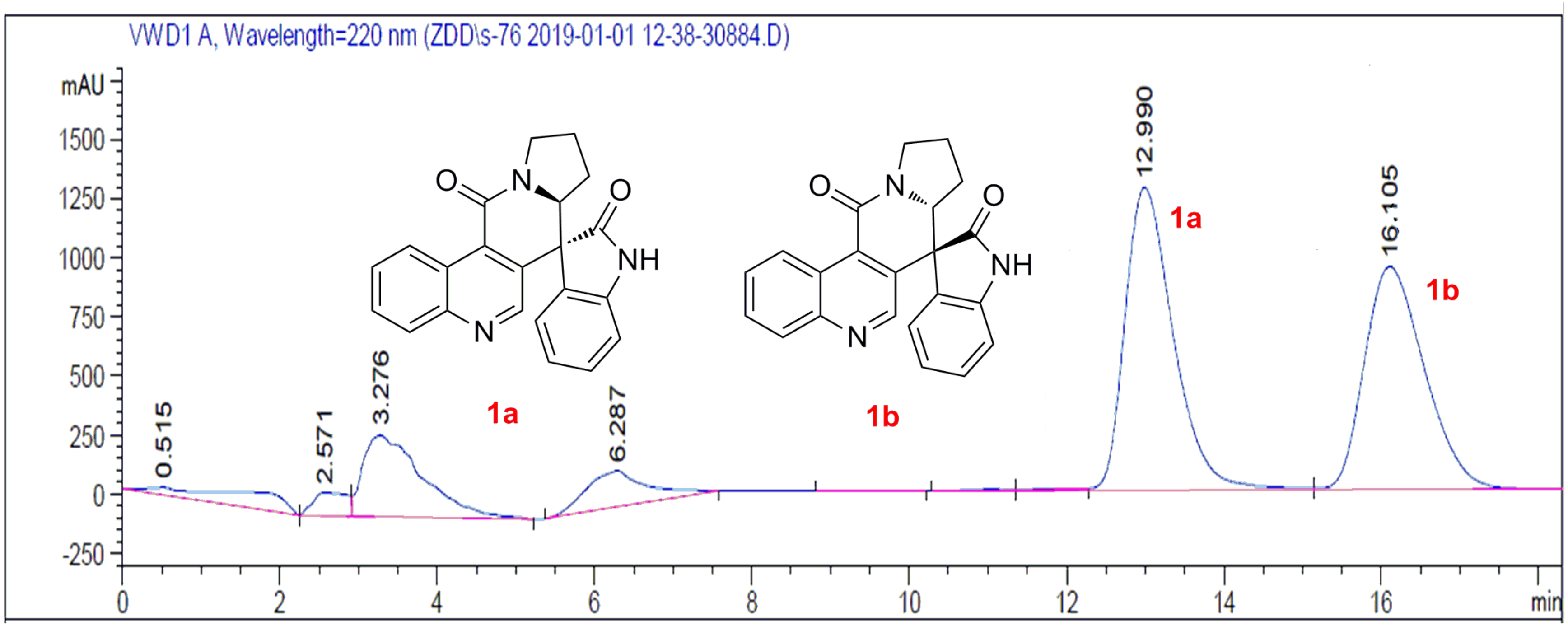

Figure S34. Chiral separation chromatography of **1**

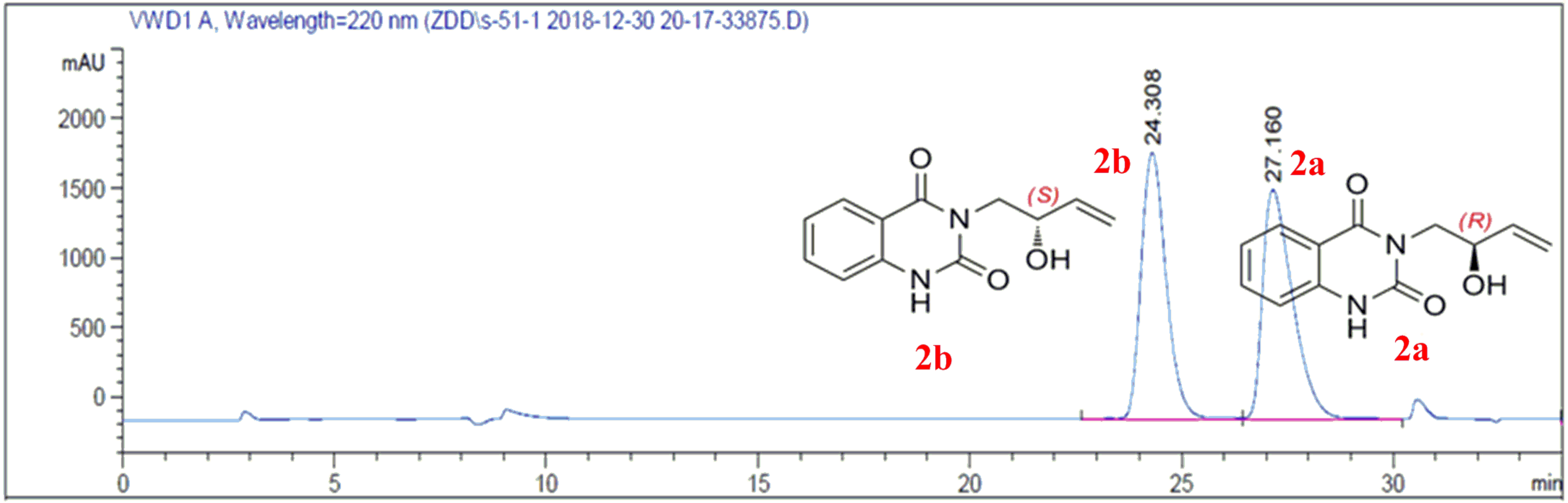

Figure S35. Chiral separation chromatography of **2**

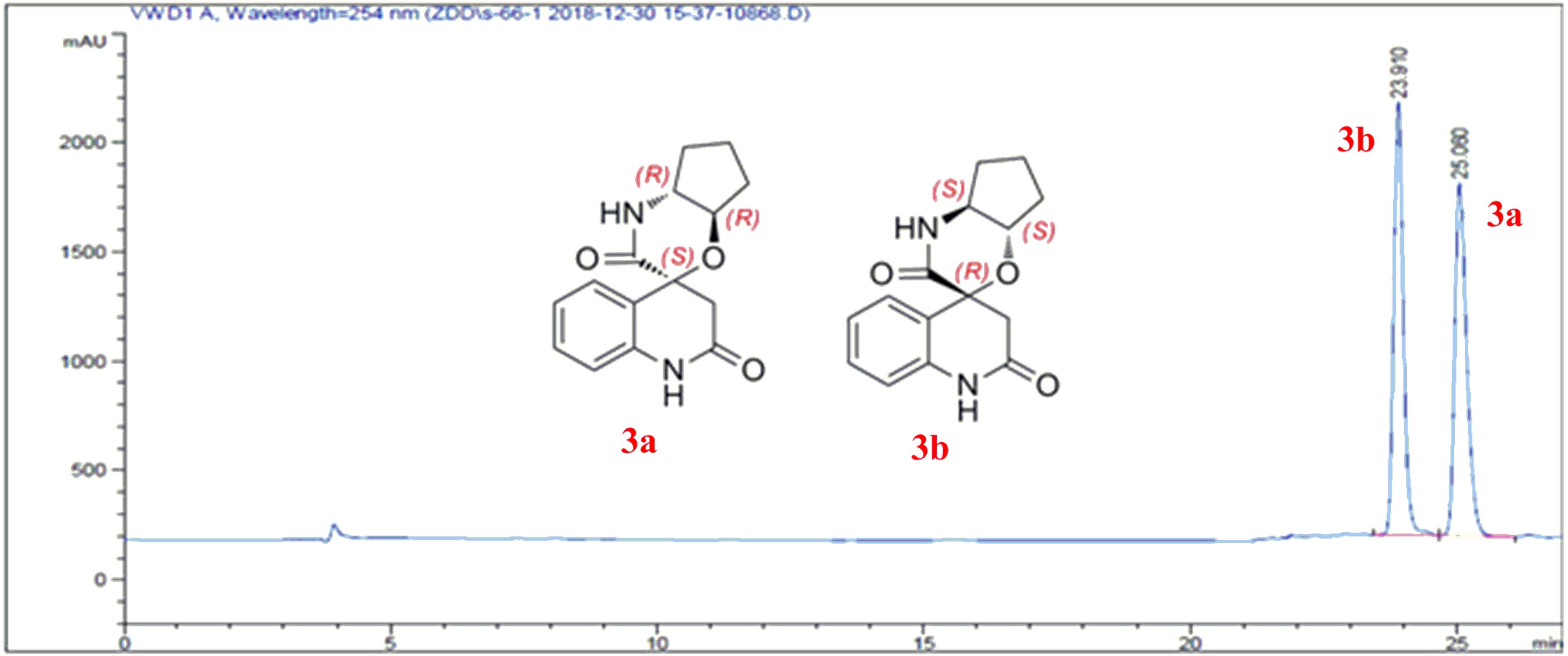

Figure S36. Chiral separation chromatography of **3**

22019060549S_0m

Table 1 Crystal data and structure refinement for 22019060549S_0m.

Identification code	22019060549S_0m
Empirical formula	$C_{25}H_{27}N_4O_7$
Formula weight	495.50
Temperature/K	130.0
Crystal system	orthorhombic
Space group	$P2_12_12_1$
a/Å	7.7008(2)
b/Å	13.3435(4)
c/Å	23.1191(7)
α/°	90
β/°	90
γ/°	90
Volume/Å^3	2375.62(12)
Z	4
ρ_{calc}g/cm^3	1.385
μ/mm^{-1}	0.856
F(000)	1044.0
Crystal size/mm^3	0.19 × 0.08 × 0.05
Radiation	CuKα (λ = 1.54178)
2Θ range for data collection/°	7.648 to 148.48
Index ranges	-9 ≤ h ≤ 8, -15 ≤ k ≤ 16, -28 ≤ l ≤ 28
Reflections collected	31880
Independent reflections	4813 [R_{int} = 0.0526, R_{sigma} = 0.0284]
Data/restraints/parameters	4813/367/347
Goodness-of-fit on F^2	1.045
Final R indexes [I>=2σ (I)]	R_1 = 0.0537, wR_2 = 0.1582
Final R indexes [all data]	R_1 = 0.0572, wR_2 = 0.1613
Largest diff. peak/hole / e Å^{-3}	0.37/-0.37
Flack parameter	0.15(7)

Figure S37. Crystallographic data of **2b**

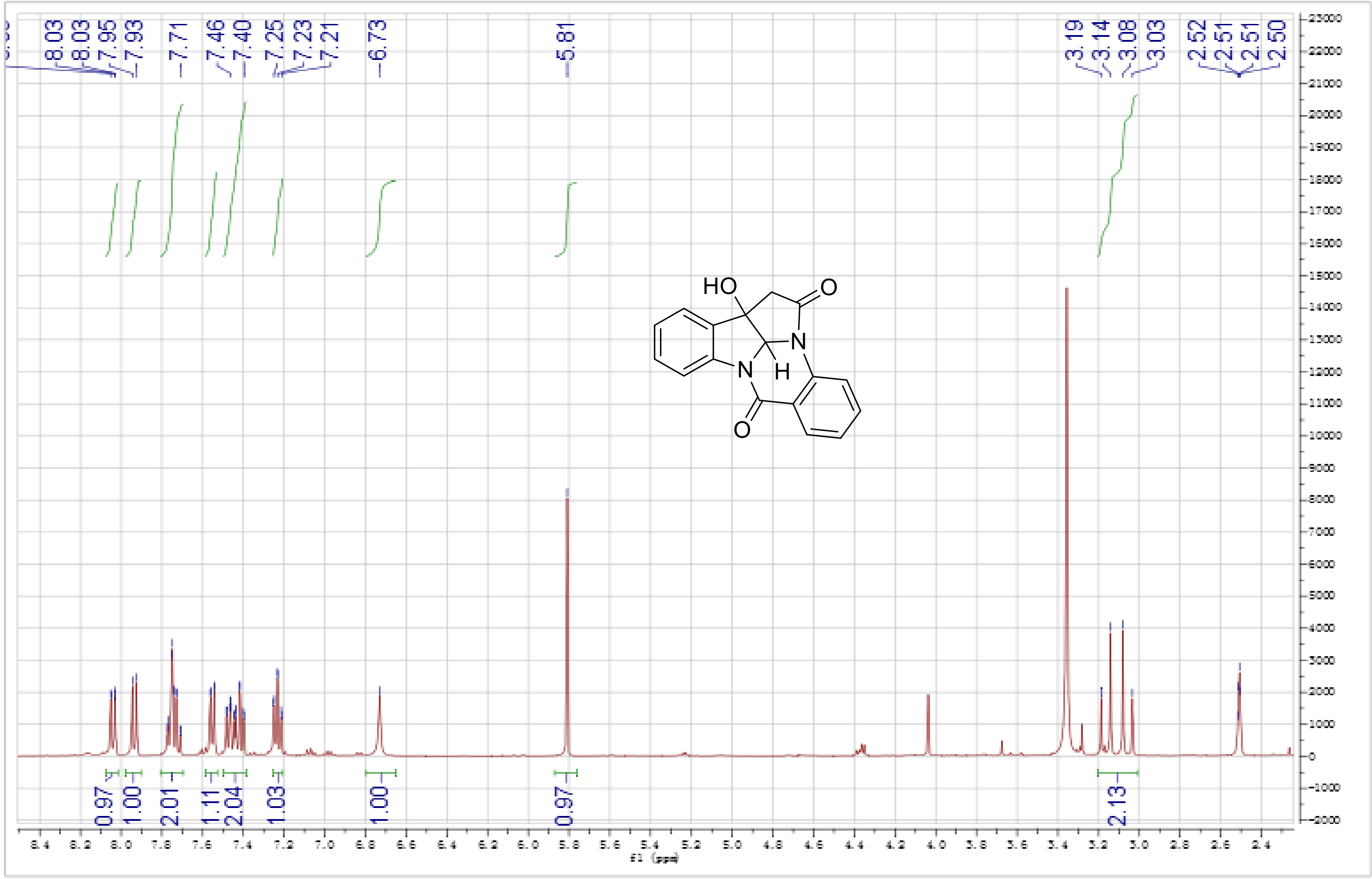

Figure S38. The ^{1}H NMR spectrum of **4a/4b** (in DMSO-d_6)

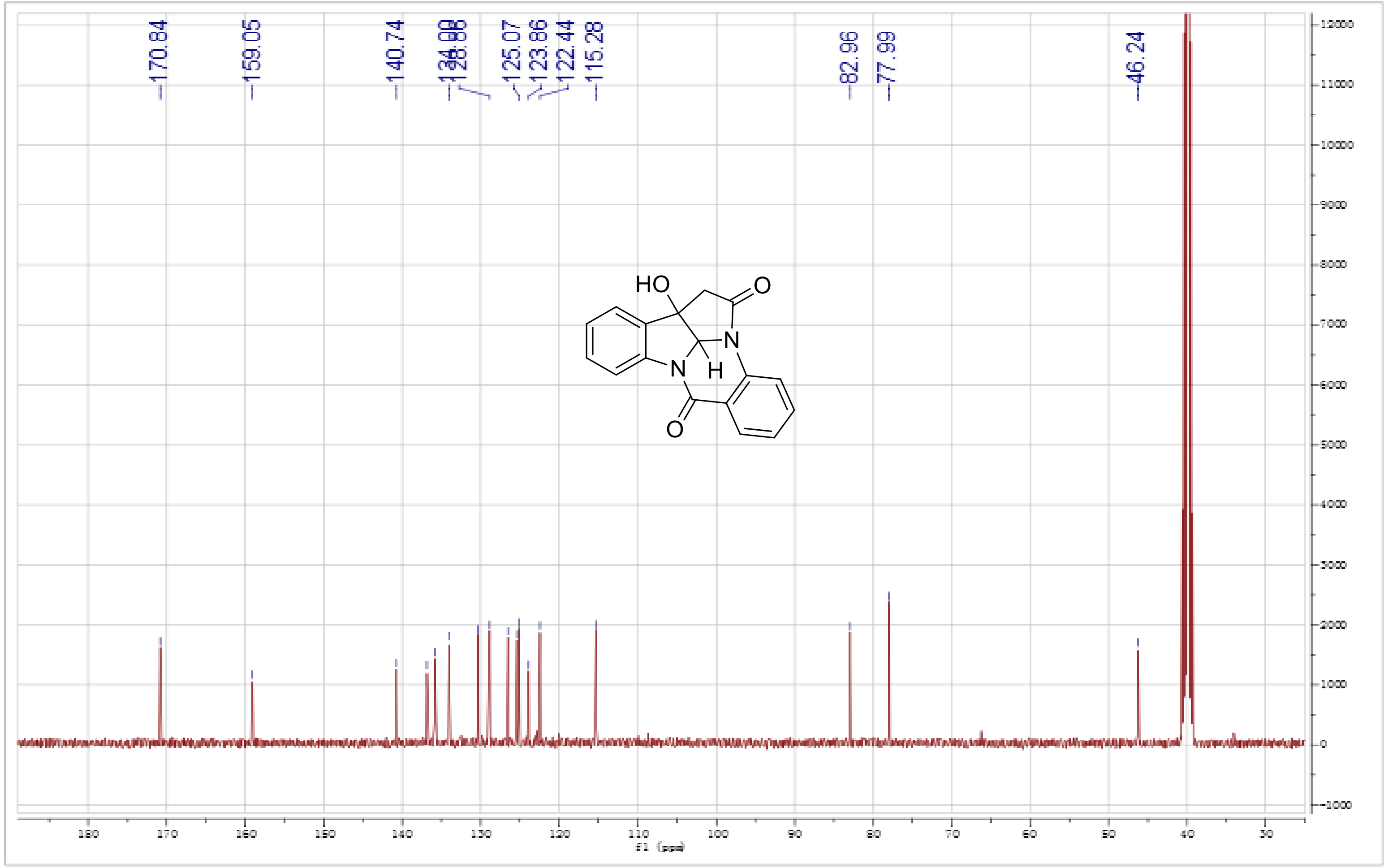

Figure S39. The ^{13}C NMR spectrum of **4a/4b** (in DMSO-d_6)

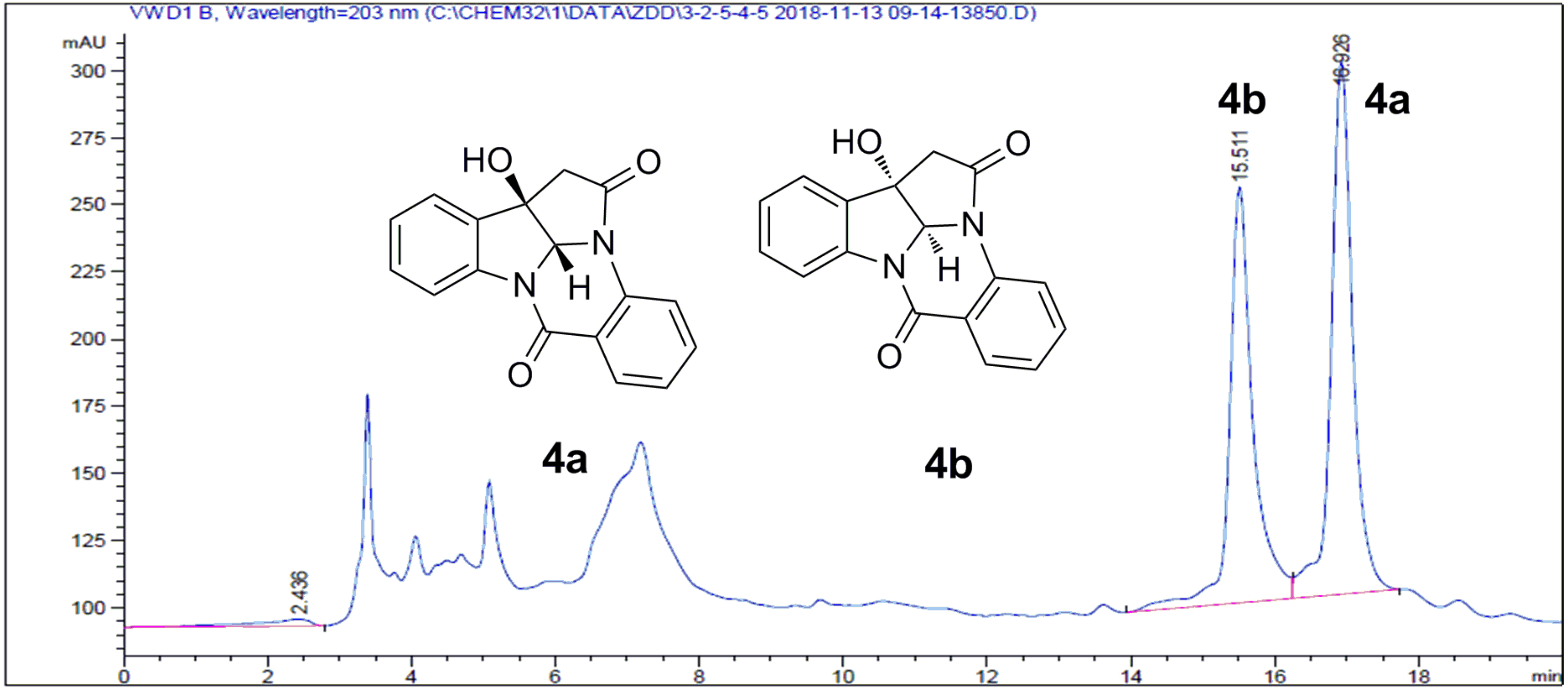

Figure S40. Chiral separation chromatography of **4**

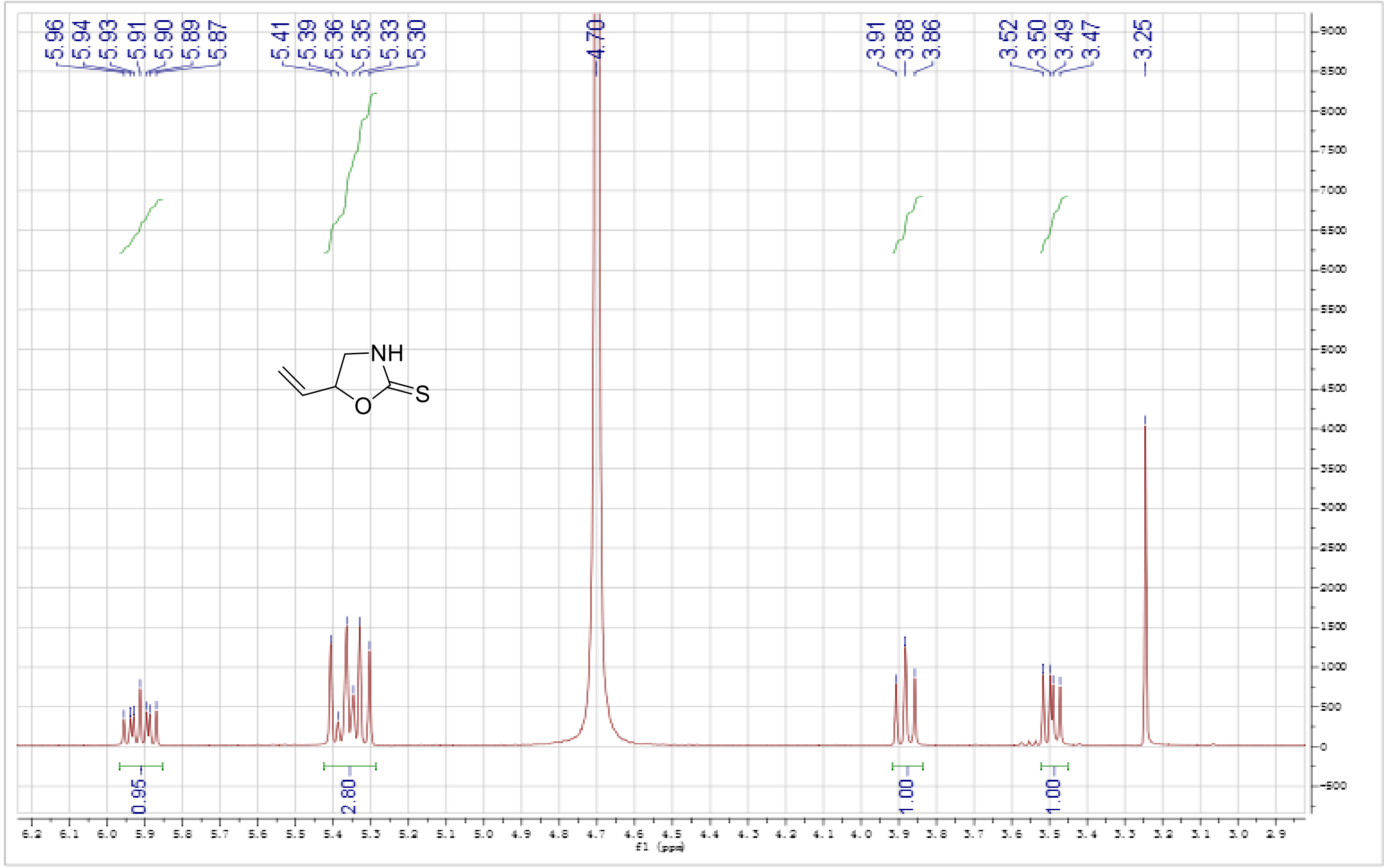

Figure S41. The ^{1}H NMR spectrum of **1a/1b** (in D_2O)

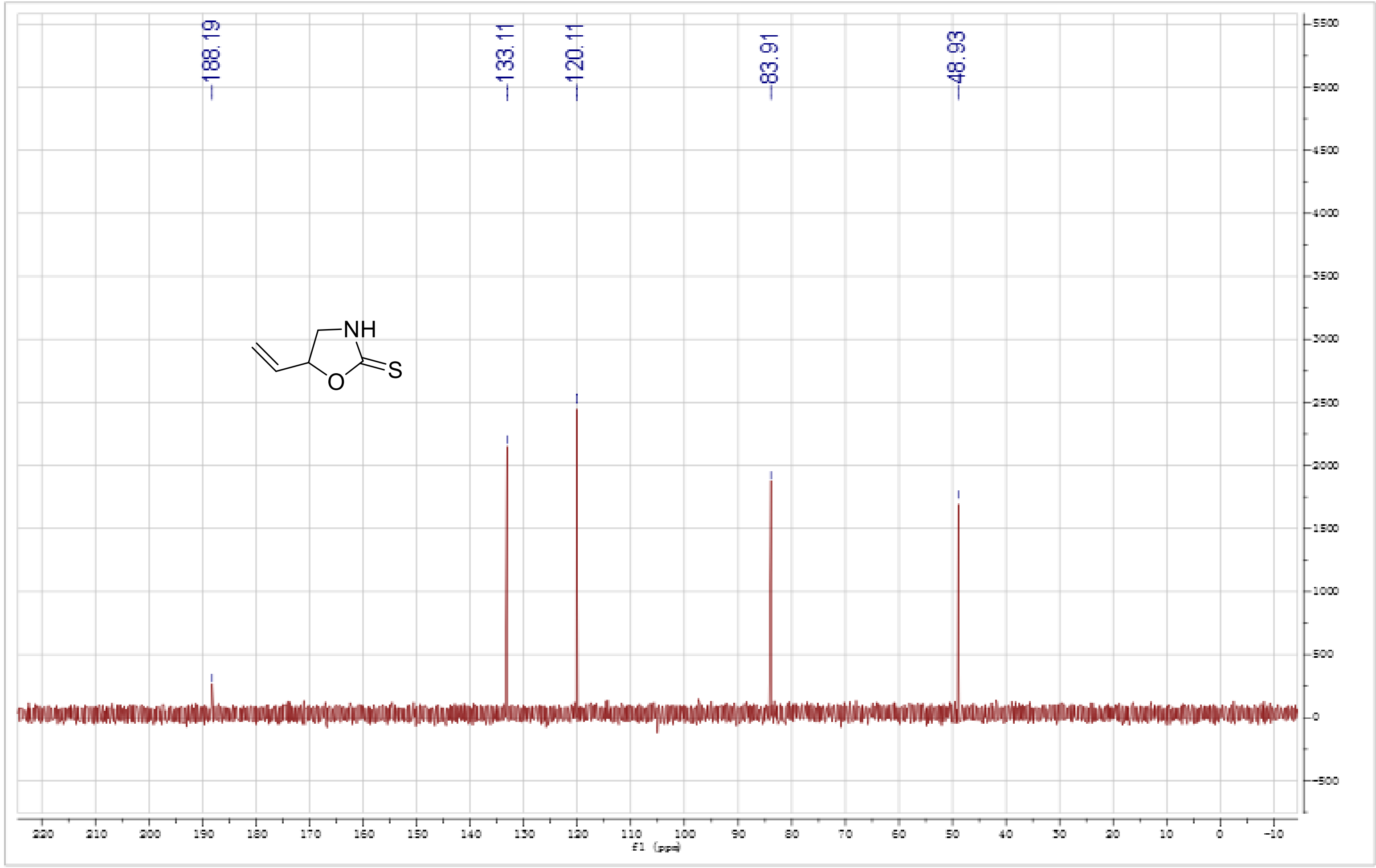

Figure S42. The ^{13}C NMR spectrum of **1a/1b** (in D_2O)

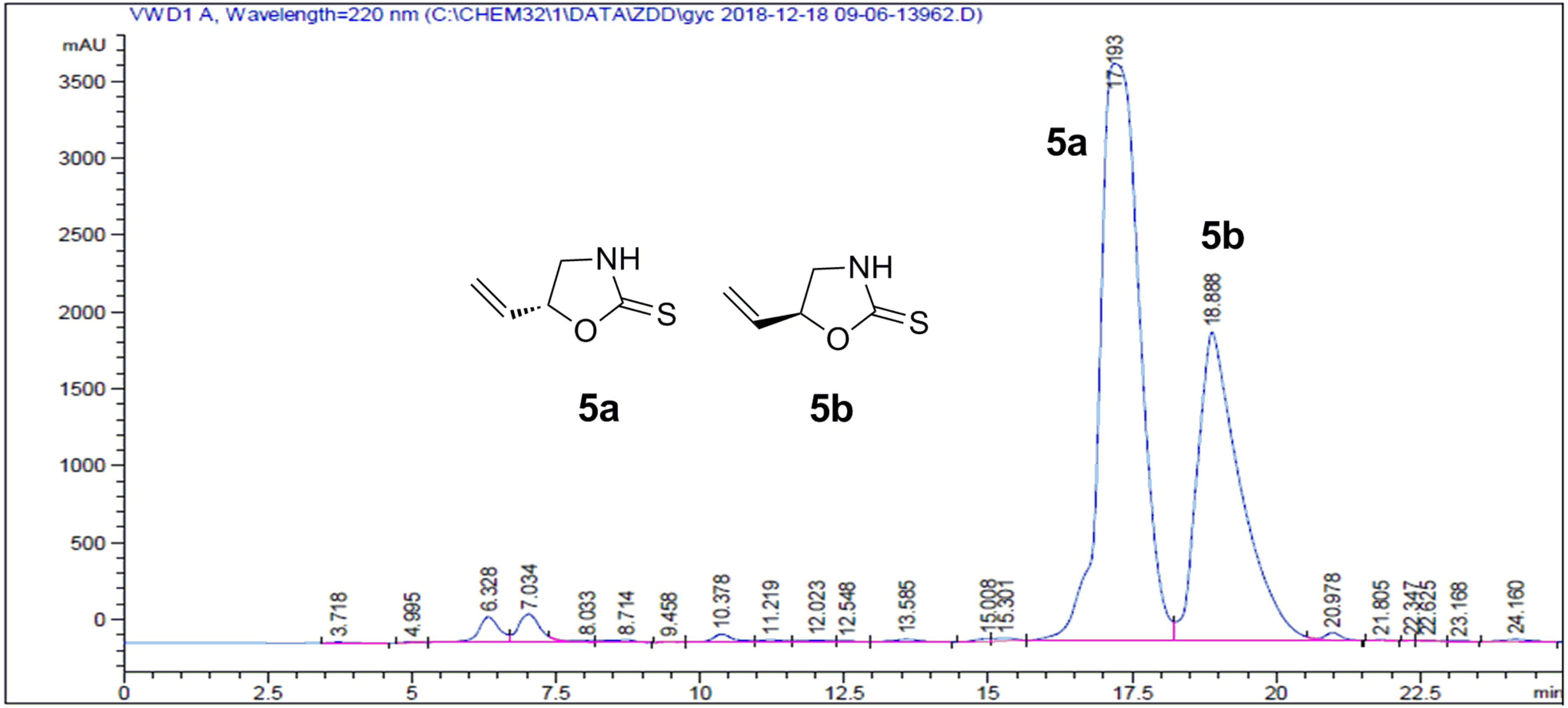

Figure S43. Chiral separation chromatography of **5**
